# Fast Sodium Ion Conductivity in Pristine Na_8_SnP_4_: Synthesis, Structure and Properties of the Two Polymorphs LT‐Na_8_SnP_4_ and HT‐Na_8_SnP_4_

**DOI:** 10.1002/anie.202419381

**Published:** 2025-04-29

**Authors:** Manuel Botta, Samuel Merk, Robert J. Spranger, Anatoliy Senyshyn, Volodymyr Baran, Vadim Dyadkin, Leo van Wüllen, Thomas F. Fässler

**Affiliations:** ^1^ TUM School of Natural Sciences Department of Chemistry Chair of Inorganic Chemistry with Focus on New Materials Technical University of Munich (TUM) Lichtenbergstraße 4 Garching D‐85748 Germany; ^2^ TUMint.Energy Research GmbH Lichtenbergstraße 4 Garching D‐85748 Germany; ^3^ Department of Physics University of Augsburg Universitätsstraße 1 Augsburg 86159 Germany; ^4^ Research Neutron Source Heinz Meier‐Leibnitz (FRM II) Technische Universität München Lichtenbergstraße 4 Garching bei München 85747 Germany; ^5^ Deutsches Elektronen Synchrotron (DESY) Notkestr. 85 Hamburg 22607 Germany; ^6^ Swiss‐Norwegian Beam Lines ESRF‐The European Synchrotron 71, Avenue des Martyrs Grenoble Cedex 9 38042 France

**Keywords:** Impedance spectroscopy, Ion conductor, Phosphide, Sodium, Structure

## Abstract

Achieving high ionic conductivities in solid state electrolytes is crucial for the development of efficient all‐solid‐state‐batteries. Considering future availability and sustainability, sodium materials hold promises for an alternative for lithium materials in all‐solid‐state batteries, due to the higher abundance. Here, we report on a sodium phosphide ion conductor Na_8_SnP_4_ with a conductivity of 0.53 mS cm^−1^ at room temperature as a pristine material. Due to the simple tetrahedral SnP_4_ structure units, Na_8_SnP_4_ has potential for optimization through aliovalent substitution as successfully applied in sulfide‐based materials. Na_8_SnP_4_ is easily accessible from exclusively abundant elements and forms a high‐ and low‐temperature polymorph, which further allows for a fundamental understanding of the structure‐property relationship. Both polymorphs are structurally characterized by synchrotron X‐ray powder diffraction and MAS–NMR spectroscopy. Ion conductivity and activation energy for ion mobility is determined by temperature dependent impedance spectroscopy and static ^23^Na‐NMR measurements. Both MEM analysis of scattering densities as well as structure determination by Rietveld methods hint for ionic motion between special Na positions in the structure and that ion migration proceeds along pathways passing triangular faces of neighboring tetrahedral and octahedral voids. The specific voids filling in the disordered HT‐phase are found to be a crucial parameter for ion migration.

## Graphical abstract

Sodium‐ion conductors show great promise for use in rechargeable solid‐state batteries due to the high natural abundance of sodium. As a pristine material, Na_8_SnP_4_ demonstrates relatively high ionic conductivity and offers significant room for further enhancement, owing to its structure built on well‐known tetrahedral building blocks. Additionally, its existence as both high‐ and low‐temperature polymorphs provides valuable insights into the structure‐property relationship.

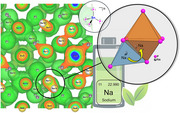

## Introduction

Lithium‐ion batteries (LIBs) are the most widely used rechargeable batteries today with applications ranging from smartphones and electric vehicles to stationary storage devices. Na‐ion batteries (NIBs) are in the focus of interest as a possible alternative for the widely used LIBs because of the higher natural abundance and lower production costs of sodium if compared to lithium. However, since Na is approximately three‐fold heavier and possesses a lower standard electrochemical potential than Li, NIBs will not easily surpass LIBs with respect to energy density and specific capacity.^[^ ^1^, ^2^, ^3^ ^]^ Nevertheless, driven by the demand on high safety, the demand for stationary energy storage system, and the implementation of metal anodes for higher capacity, the shift from liquid electrolytes to inorganic solid electrolytes attracted tremendous research attentions in recent years for the design of solid‐state electrolytes (SSEs) also in NIBs.^[^ ^4^, ^5^ ^]^

Compared to the huge number of recent publications dealing with Li^+^ solid electrolyte materials, the research performed on Na^+^ solid electrolyte materials are rather narrow due to paucity of promising fast Na‐ion conducting materials. The oldest and most well‐investigated example of oxide‐based Na^+^ solid electrolyte materials is Na‐β’’‐Al_2_O_3_ which exhibits very high ionic conductivity if prepared with high crystallinity.^[^ ^6^, ^7^ ^]^ However, the complicated preparation prevents large‐scale usage. The investigation on sulfide based materials started with pristine cubic Na_3_PaS_4_ in 1992^[^ ^8^ ^]^ which exhibited a conductivity of 0.2 mS cm^−1^ as a glass‐ceramic electrolyte which could be improved to 0.46 mS cm^−1^ using high‐purity starting materials.^[^ ^9^, ^10^ ^]^ Further investigations on this compound class led to the derivatives Na_3_PSe_4_^[^ ^11^ ^]^ with a conductivity of 1.2 mS cm^−1^ and Na_3_SbS_4_^[^ ^12^ ^]^ with 1–3 mS cm^−1^. The high ionic conductivities led to deeper investigations of the material in the form of theoretical calculations, which revealed that halide doping could further enhance Na‐ion mobility through the creation of vacancies. Indeed, Na_3−x_PS_4−x_Cl_x_ (*x* = 6,25%) reaches a conductivity of 1.14 mS cm^−1^.^[^ ^13^ ^]^ Waghmare and co‐workers computed the sodium derivative of the fast Li‐ion conductor Li_10_ *Tt*P_2_S_12_ (Tt = Si, Ge, Sn) through the means of density functional theory (DFT)^[^ ^14^ ^]^ and obtained a theoretical conductivity of 4.7 mS cm^−1^ for Na_10_GeP_2_S_12_. Richards et al. confirmed these results and expanded the calculations on the whole system of Na_10_ *Tt*P_2_S_12_ (Tt = Si, Ge, Sn) showing a trend of increasing conductivities in the order of Si→Ge→Sn.^[^ ^15^ ^]^ They also attempted to synthesize “Na_10_SnP_2_S_12_” but failed to achieve a phase pure product. Two years later, the compound Na_11_Sn_2_PS_12_ was reported and exhibited a very high ionic conductivity of 3.7 mS cm^−1^.^[^ ^16^, ^17^ ^]^ So far, the highest sodium ion conductivities were achieved by substitution of SbS_4_^3−^ by WS_4_^2−^ in Na_3_SbS_4_ resulting in compounds like Na_2.88_Sb_0.88_W_0.12_S_4_^[^ ^18^ ^]^ or Na_2.9_Sb_0.9_W_0.1_S_4_^[^ ^19^ ^]^ with conductivity values of 32 mS cm^−1^ and 41 mS cm^−1^, respectively, at room temperature. All compounds possess discrete tetrahedral building units, that allowed for the optimization.

We have recently introduced phosphide‐based materials that show high lithium‐ion conductivity comparable to the state‐of‐the‐art materials. The compounds Li_8_ *Tt*P_4_ (*Tt *= Si, Ge, Sn)^[^ ^20^, ^21^, ^22^ ^]^ and Li_9_ *Tr*P_4_ (*Tr *= Al, Ga, In)^[^ ^23^, ^24^, ^25^ ^]^ all comprise of discrete [*Tt*P_4_]^8−^ and [*Tr*P_4_]^9−^ tetrahedra as building units balanced by surrounding Li^+^ ions. The compounds Li_14_ *Tt*P_6_ (*Tt *= Si, Ge, Sn)^[^ ^26^, ^27^ ^]^ additionally possess P^3−^ units and can incorporate even more charge carriers. Most interestingly, all compounds offer a clear structural relation based on a *ccp* arrangement of phosphorus as well as diverse chemical and electrochemical properties. This multitude of compounds enables detailed investigations on structure‐property relationships for phosphide‐based ion conductor materials such as, for instance, showing that the degree of filling and ordering of tetrahedral and octahedral voids highly impacts on the ionic conductivity or that ion diffusion occurs preferably between face‐sharing tetrahedral and octahedral sites.

Interestingly, reports on materials in the system of sodium phosphidotetrelates are scarce. The few known representatives of sodium ion conducting phosphidotetrelates occur all in the Na‐Si‐P system, with the six known compounds Na_19_Si_13_P_25_, Na_23_Si_37_P_57_, Na_23_Si_28_P_45_, and LT‐ as well as HT‐NaSi_2_P_3_ forming three‐dimensional networks. The ionic conductivity values are in the range of 1.76 x 10^−9^ mS cm^−1^ (Na_19_Si_13_P_25_) to 0.4 mS cm^−1^ (HT‐NaSi_2_P_3_) along with low activation energies of 0.23 eV and 0.25 eV for LT‐ and HT‐NaSi_2_P_3,_ respectively, thus reaching the range of sulfur‐based compounds.^[^ ^28^ ^]^ We recently reported on the compounds Na_8_GeP_4_^[^ ^29^ ^]^ and Na_7_TaP_4_^[^ ^30^ ^]^ where we managed to transfer structural properties of discrete tetrahedral building blocks observed in above mentioned lithium compounds to a sodium compound. The compounds exhibit isolated [GeP_4_]^8−^ and [TaP_4_]^7−^ tetrahedra, respectively, with Ge and Ta atoms located in tetrahedral voids of the *ccp* of P atoms and crystallization with a different ordering of tetrahedra compared to the lithiated compounds. This seemingly induced a high electronic conductivity in Na_8_GeP_4_ that might overtake ionic conduction and made Na_8_GeP_4_ an electron conductor, whereas Na_7_TaP_4_ showed no Na‐ion or electron conduction at all.

We thus attempted to expand the compound class from Ge to the heavier homologue Sn, as Sn‐based lithium compounds^[^ ^21^ ^]^ have shown to exhibit higher ionic conductivities. By adapting the synthesis protocols for Li compounds to its heavier Na homologue we succeeded in finding two polymorphs of the new sodium phosphidostannate Na_8_SnP_4_, which now comprises of simple building blocks in the form of discrete SnP_4_ units. The materials are accessible in both gram scale and high purity. Rietveld refinement against synchrotron powder‐XRD measurements at RT was performed to characterize the structure and temperature dependent measurements allowed for the investigation of thermal behavior of the polymorphs while MEM analysis of the refinement data gave first insight on possible conduction pathways. MAS–NMR experiments confirm the structure solutions. Impedance spectroscopy was performed to determine ionic and electrical conductivities of the materials. Temperature dependent impedance and static ^23^Na‐NMR spectroscopy were used to determine the activation energies of Na^+^ ion mobility. Analysis of synchrotron XRD data by the MEM was applied to get an insight in the ion motion pathways.

## Results and Discussion

### Synthesis and Crystal Structure of LT‐Na_8_SnP_4_ and HT‐Na_8_SnP_4_

**Figure 1:**
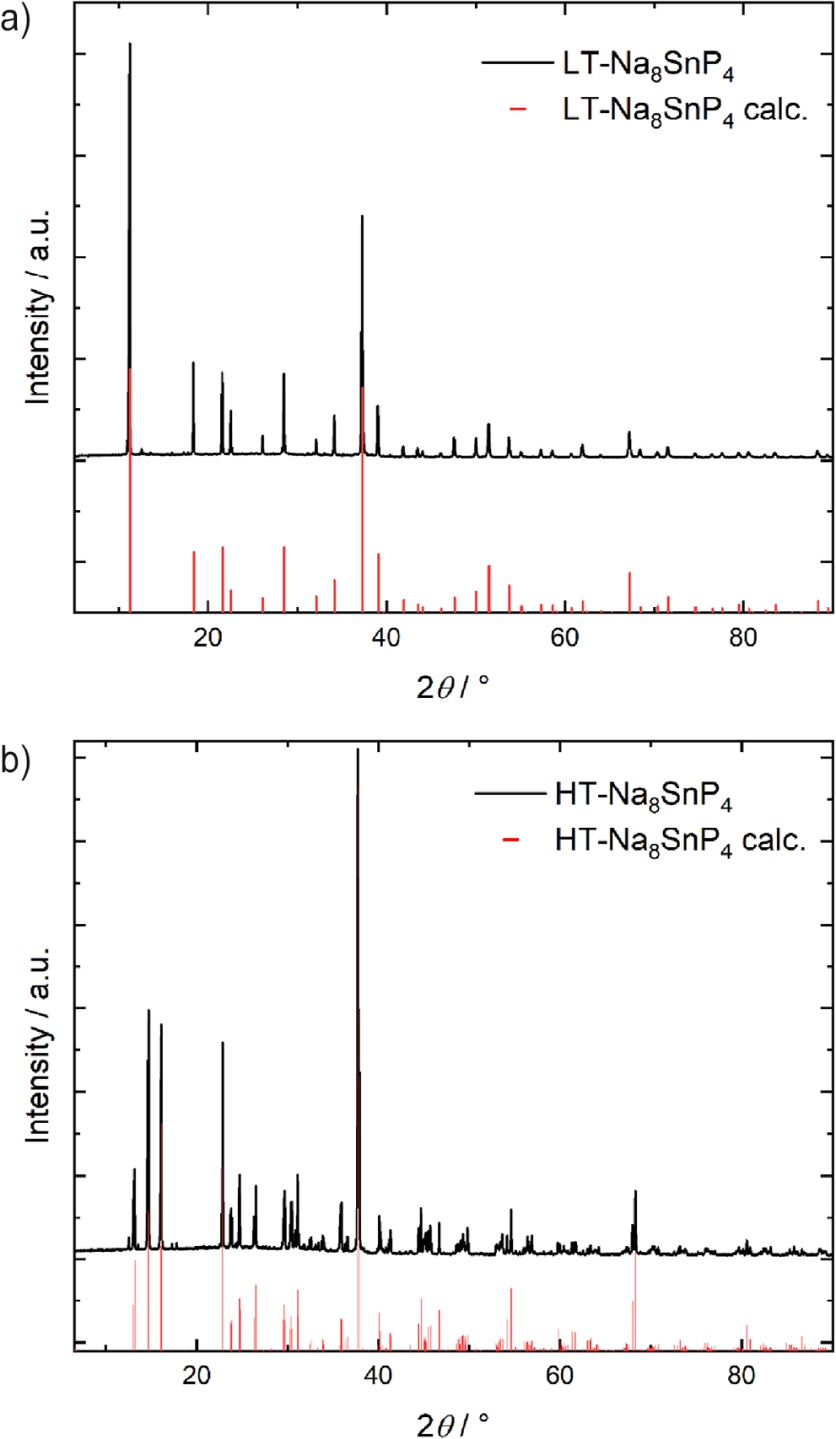
Experimental powder X‐ray diffraction pattern of the products of a stoichiometric ratio of “Na_8_SnP_4_” after ball milling and subsequent annealing at 600 °C in niobium ampoules. a) with slow cooling to RT, b) with rapid cooling in an ice/water bath. Minor extra diffraction signals in the range of 10–20° can be attributed to impurity of <3% Na_10_Sn_2_P_6_.

The two polymorphs low temperature (LT)‐Na_8_SnP_4_ and high temperature (HT)‐Na_8_SnP_4_ were obtained in gram scale from the elements via a two‐step synthesis protocol. For this, the elements sodium, tin, and red phosphorous are, according to the formal stoichiometry of “Na_8_SnP_4_”, intensively homogenized using a planetary ball mill. The intermediate powdered product is further annealed at 873 K and subsequently either slowly cooled to room temperature to receive the (LT) phase or rapidly cooled by quenching to room temperature in order to receive the (HT) modification, respectively. The obtained powder patterns are shown in Figure 1.

The structural determination of both phases was performed by Rietveld analysis using synchrotron powder X‐ray diffraction data. The resulting Rietveld analysis of both phases is given in the Supporting Information (Figure S2) and shows that both phases are obtained in high purity of >97(1) wt.‐% with the minor impurity <3(1) wt.‐% of Na_10_Sn_2_P_6_, which we found also to be the decomposition product at higher temperatures as shown below. Atomic coordinates and isotropic displacement parameters are listed in Tables S3,S4 in the Supporting Information. Additionally, selected interatomic distances and angles of both LT‐Na_8_SnP_4_ and HT‐Na_8_SnP_4_ are given in the Supporting Information (Tables S5–S8).

**Figure 2:**
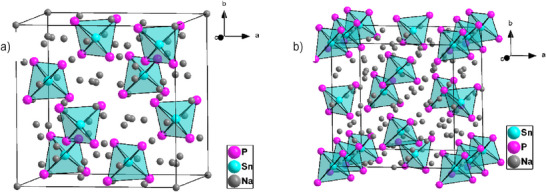
Crystal structure of a) LT‐Na_8_SnP_4_ and, b) HT‐Na_8_SnP_4_ with view in *c* direction. Sodium atoms are located surrounding the [SnP_4_]^8−^ tetrahedra in tetrahedral and octahedral voids of the phosphorous *ccp*. The Na, Ge, and P atoms are drawn in grey, blue, and pink, respectively. The [SnP_4_]^8−^ tetrahedra are highlighted as turquoise polyhedra.

LT‐Na_8_SnP_4_ crystallizes in the cubic space group *Fd* $\bar{3}$ *m* (no. 227) with a lattice parameter of *a* = 13.62240(3) Å at 297K and is symmetrically connected to the antiflouride structure being structurally isotypic to Na_8_GeP_4_, thus adopting the Na_8_SnSb_4_ structure type.^[^ ^31^ ^]^ Furthermore, it is homeotypic to the related lithiated compounds Li_8_ *Tt*P_4_ (*Tt *= Si, Ge, Sn).^[^ ^20^, ^21^, ^22^ ^]^ Details of the structure refinement are given in Table 1. LT‐Na_8_SnP_4_ comprises of four crystallographically independent and fully occupied atom positions Sn1, P1, Na1, and Na2 (Figure 2a), that form tetrahedral [SnP_4_]^8−^ units separated by eight Na^+^ ions per formula unit. The P atoms form a slightly distorted cubic‐close atom arrangement (*ccp*) with the P atoms slightly shifted towards the Sn‐atom that occupies a tetrahedral void retaining an ideal tetrahedron with a P‐Sn‐P angle of 109.47°. The Sn─P distance of 2.5886(8) Å is within the characteristic range of Sn─P bonding interactions in related compounds such as Na_10_Sn_2_P_6_ (2.50–2.59 Å),^[^ ^32^ ^]^ Li_8_SnP_4_ (2.54–2.55 Å)^[^ ^21^ ^]^ or NaSnP (2.59 Å).^[^ ^33^ ^]^ All edges of the [SnP_4_]^8−^ tetrahedra are capped by Na2 while all tetrahedral faces are capped by Na1 with Na─P distances in the range of 2.946(2) Å to 3.208(1) Å (Supporting Information Table S5). Besides Sn1 (Wyckoff site 8a corresponding to 12,5% of the tetrahedral voids), Na2 (Wyckoff site 48f) fills 75% of the tetrahedral voids and 12.5% (Wyckoff site 8b) remain vacant. The residual Na atoms occupy 50% of the octahedral voids (Na1 at 16c) while the remaining 50% of the octahedral sites (16d) stay empty. For Na1 and Na2 a perfect NaP_6_ octahedron and a slightly distorted NaP_4_ tetrahedron, respectively is formed (Figure S3, Supporting Information).

**Table 1:** Refinement data of Rietveld refinement of powder synchrotron XRD‐data on LT‐Na_8_SnP_4_ at 293K and on HT‐Na_8_SnP_4_ at 293K.

Empirical Formula	LT‐Na_8_SnP_4_	HT‐Na_8_SnP_4_
*T* / K	293	293
Formula weight / g mol^−1^	426.5	426.5
Space group (no)	*Fd* $\bar{3}$ *m* (227)	*P* $\bar{4}$2*c* (112)
Unit cell parameters / Å	*a *= 13.62240(3)	*a *= 13.42471(5)
		*c *= 13.53135(7)
*Z*	8	8
*V* / Å^3^	2527.90(1)	2438.66(2)
*ρcalc*. / g cm^−3^	2.24128	2.319
θ range / deg	0.0044–21.592	0.046–35.569
*Rp*	1.3224	4.2601
*Rwp*	1.9986	6.5581
*Rexp*	1.0090	0.0370
*R_Bragg_*	6.41	5.45
*R_f_*	3.63	5.85
*Depository no*.	CSD‐2311948	CSD‐2311919

HT‐Na_8_SnP_4_ crystallizes in the space group *P* $\bar{4}$2*c* (no. 112) with lattice parameters of *a* = 13.42471(5) Å and *c*  =  13.53135(7) Å making this the first compound with tetragonal structure in the group of phosphidotetrelates. The details of the structure refinement are given in Table 1 as well as in the Supporting Information (Tables S7,S8). The structure can be deduced through symmetry degradation from the related cubic lithium compound β‐Li_8_SnP_4_^[^ ^21^ ^]^ into the tetragonal space group *P* $\bar{4}2$ *c* by elongation of the c‐axis of a cubic cell that results in the formation of a tetragonal system (Figure S8, Supporting Information). Like the LT‐Phase, HT‐Na_8_SnP_4_ also consists of isolated highly charged [SnP_4_]^8−^ tetrahedra that are charge balanced by the corresponding number of Na^+^ ions. The structure comprises 23 independent crystallographic positions (Sn1Sn3, P1‐P4 and Na1‐Na16) of which all Sn and P position are fully occupied. The P atoms form again a distorted *ccp* where they are slightly shifted towards the Sn‐atom positions that are located in tetrahedral voids. In contrast to LT‐Na_8_SnP_4_, in which specific tetrahedral and octahedral voids remain vacant, in HT‐Na_8_SnP_4_ all remaining tetrahedral and all octahedral voids are occupied by Na atoms. In summary, 1/8^th^ of the tetrahedral voids are occupied by Sn while the remaining 7/8^th^ of the tetrahedral voids are occupied by Na (Na1 to Na12). Out of these tetrahedral coordinated Na positions, Na9, Na11, and Na12 are partially occupied with S.O.F values of 0.78, 0.57, and 0.64, respectively, resulting in an average of 82.6% sodium occupation in 7/8^th^ of the voids. The total average occupation of all tetrahedral voids including Sn therefore is 84.7%. The remaining four Na positions (Na 13‐Na16) are distributed over octahedral voids, with all positions partially occupied with S.O.F values of 0.21, 0.62, 0.70, and 0.43 for Na13, Na14, Na15, and Na16, respectively, resulting in a total occupation of 49% of all octahedral voids. The Sn‐Atoms are surrounded by four P‐Atoms each: four P4 and four P3 atoms surround Sn1 and Sn3, respectively, whereas two P3 and two P1 surrounded Sn2. The [SnP_4_]^8−^ tetrahedra exhibits various degrees of deviation from the ideal tetrahedron angle and P‐Sn‐P angles range from 109.31° to 110.0° for Sn1 and Sn3. For Sn2 slightly larger deviations in the range of 101.3° to 118.32° are present. These deviations correlate with Sn─P distances of 2.55 Å and 2.57 Å for Sn1 and Sn3, respectively, and longer distances (2.67 Å and 2.74 Å) for Sn2. This situation hints for a disorder of the (Sn2)P_4_ tetrahedron, however, the crystallographic information by powder X‐ray diffraction data is limited. Full occupation of the P and Sn atoms sites results within standard deviations in an electron precise sum formula Na_8_SnP_4_. The data are also consistent with the MAS–NMR data (see below).

**Figure 3:**
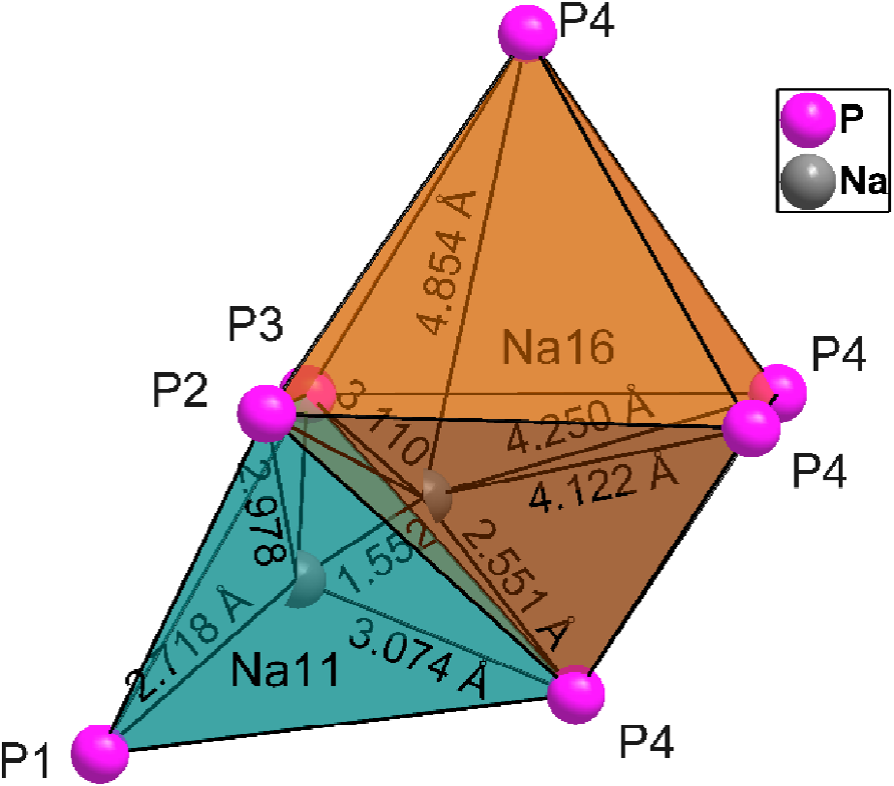
Visualization of coordination around Na11 and Na16 in HT‐Na_8_SnP_4_. Observable is the very high distortion of the octahedral Na16 atom position towards the Na11 tetrahedra indicating a possible pathway for ion migration.

In strong contrast to the LT phase, the Na atoms are distributed over twelve symmetrically independent tetrahedral and four octahedral voids formed by the P atoms. A more detailed structure description including representations of the coordination polyhedra is given in the Supporting Information, Figure S6). While octahedral Na positions Na13Na15 were refined with fixed position, Na16 could be refined without restrictions. Na16 is strongly shifted from the center of the slightly distorted octahedron formed by P2, P3, and P4 atoms towards the triangular face of the octahedron that shares face with the neighboring tetrahedron hosting Na11. Both positions are thus partially occupied and the S.O.F is constrained (Figure 3). Similar distortions and shifts towards the triangular planes as well as splitting of alkali atom positions in neighboring voids has been observed in Li_9_AlP_4_^[^ ^25^ ^]^ and sulfide‐based ion conductors,^[^ ^34^ ^]^ supporting the hypothesis of ion movement through shared faces of tetrahedral and octahedral sites. Furthermore, high isotropic values of the octahedral Na‐Sites support the concept of high disorder at these positions. For more details see Supporting information.

**Figure 4:**
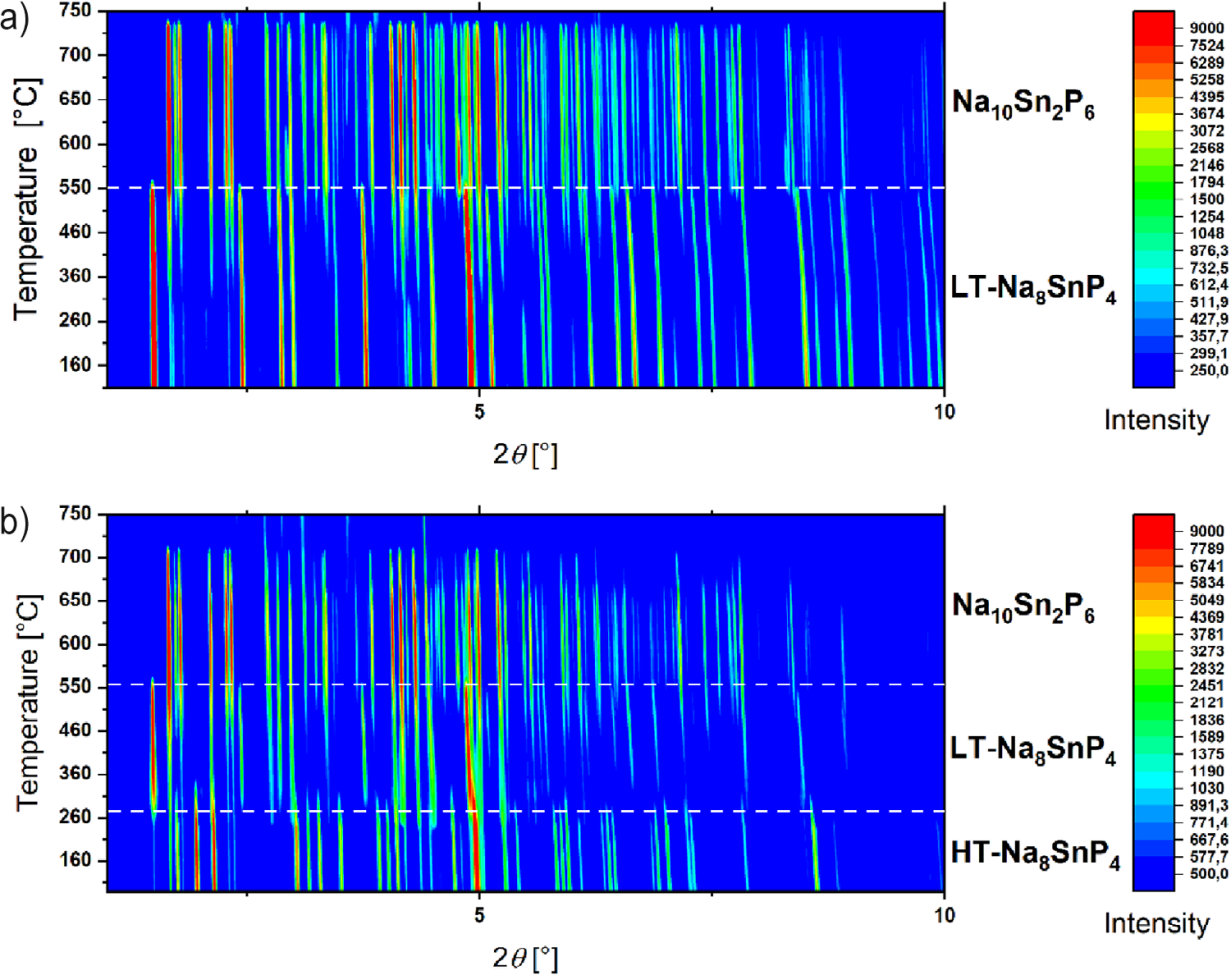
2D plot of the temperature dependent synchrotron powder X‐Ray diffraction data from 100 °C to 750 °C in a 2θ range from 0.5 to 10° (the wavelength was set to 0.20707 Å). Samples were measured in a sealed quartz capillary. a) Measurement with LT‐Na_8_SnP_4_ as starting material. b) Measurement with HT‐Na_8_SnP_4_ as starting material.

Temperature dependent synchrotron powder X‐ray diffraction measurements in the range between 30 °C and 750 °C were recorded in steps of 10 °C up to 540 °C followed by 5 °C steps to study the phase transition. Upon heating LT‐Na_8_SnP_4_ a transition at around 550 °C the vanishing of reflections at 1.5, 2.5, and 3.8°2θ indicates the loss of the cubic symmetry and formation of Na_10_Sn_2_P_6_ is observed (Figure 4a). By heating the high temperature polymorph starting at ambient temperature, the formation of LT‐Na_8_SnP_4_ is observed at 260 °C, and agrees with the fact that HT‐Na_8_SnP_4_ is meta‐stable at room temperature. Formation of Na_10_Sn_2_P_6_ occurs again at higher temperatures (Figure 4b). Na_10_Sn_2_P_6_ has also been observed as side‐product during the optimization of the synthetic protocol. The observed transition temperatures are in good accordance with DSC measurements that show two endothermic signals at 500 and 550 °C. The lack of an observed phase transition from LT‐ to HT‐Na_8_SnP_4_ indicates kinetic hindering on the time scale of the DTA experiment, as the HT polymorph is only obtained at 12h annealing time and rapid cooling. Similarly, the decomposition of Na_8_GeP_4_, to Na_10_Ge_2_P_6_ was also observed.^[^ ^29^ ^]^

**Figure 5:**
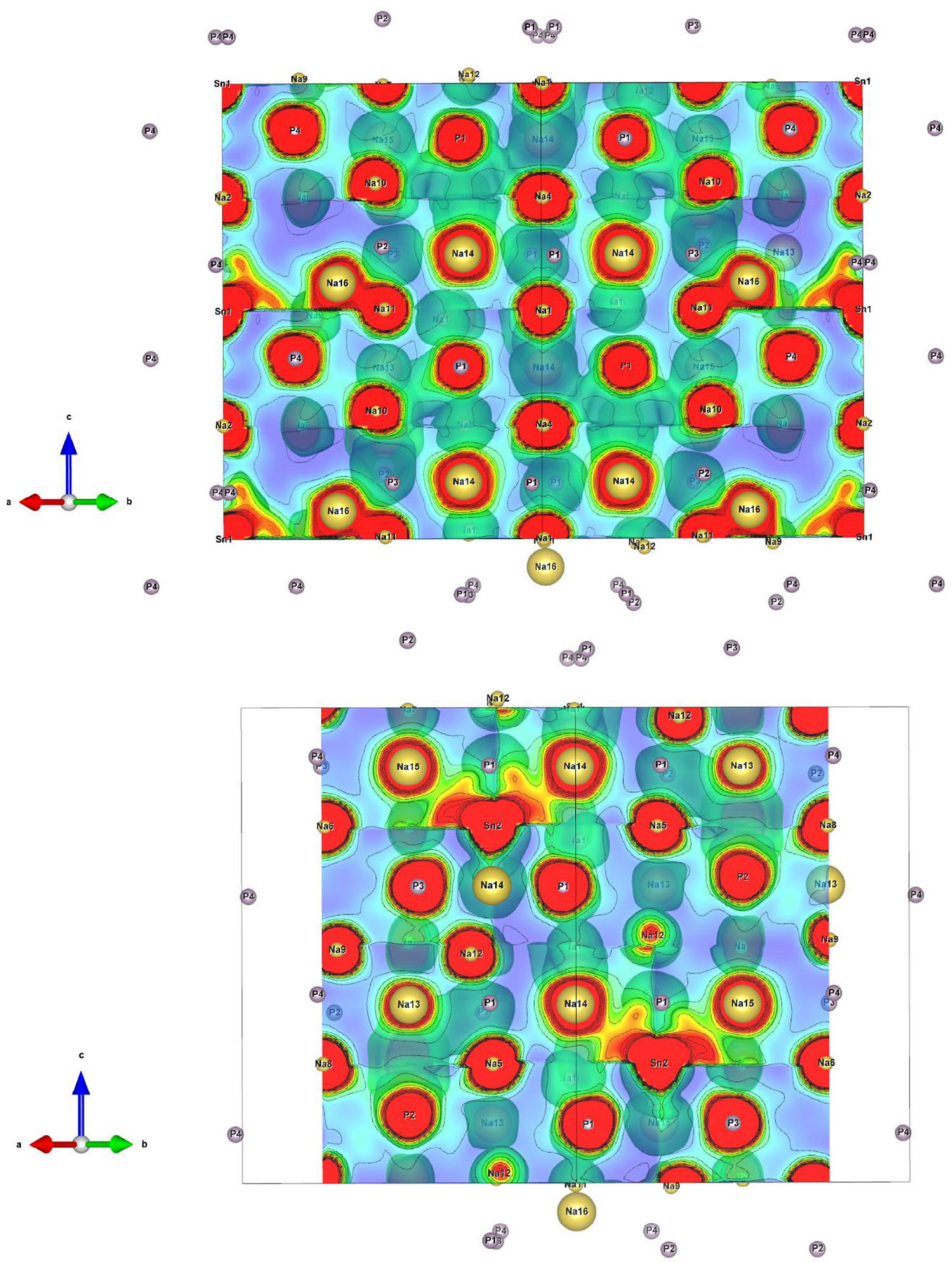
Cross section HT‐Na_8_SnP_4_ in *b*‐direction at a height of 10 Å with MEM analysis based on the temperature dependent synchrotron data The section colour code corresponds to a range from 0.4 to 2.0 with a step of 0.01 eÅ^−3^. The equi‐density contour lines are drawn from ‐1.0 to 1.0 with an interval of 0.01 eÅ^−3^.

Utilizing the temperature dependent synchrotron data, HT‐Na_8_SnP_4_ was analyzed by means of the maximum entropy method (MEM). Cutoff sections of the obtained electron density distribution in the 110‐plane at *d*  =  1 and *d*  =  0.76 are displayed in Figure 5a,b, respectively. A full cell with depicted electron charge distribution is given in the Supporting Information (Figure S9). The electron density around the Sn atoms shows clear deviations from a spherical shape with spikes towards P‐positions indicating a covalent bonding situation and supporting the model of [SnP_4_]^8−^ building units. Further “spikes” towards the neighboring Na sites supports the assumption about high disorder in these octahedral sites. Investigation of electron density distribution around Na positions is proven difficult, as an excessive overlap is observable with Sn an P electron densities hindering the determination of possible pathways for ionic motion. For the majority of the positions, the electron density distribution around Na is visible as spherical surface, leaving no hints for ionic movement. An exception are Na11 and Na16 where an overlap can be observed between each of the positions. This can be interpreted as a hint for ionic exchange in this area. The overlap is located between atoms in face‐sharing tetrahedral (Na11) and octahedral site (Na16) and thus agrees with the results from the crystallographic date of HT‐Na_8_SnP_4_ as shown in Figure 3. The results corroborate migration paths observed in the related Li‐compounds where ion migration occurs via tetrahedral to the neighboring octahedral voids through common triangular faces.^[^ ^21^, ^22^, ^23^ ^]^ In summary, the MEM analysis is in agreement with the structure determined by Rietveld refinement and gives first hints on ionic motion that is comparable to the related Li‐compounds of the system.

### ^23^Na, ^31^P, and ^119^Sn Solid‐State MAS–NMR Measurement

**Figure 6:**
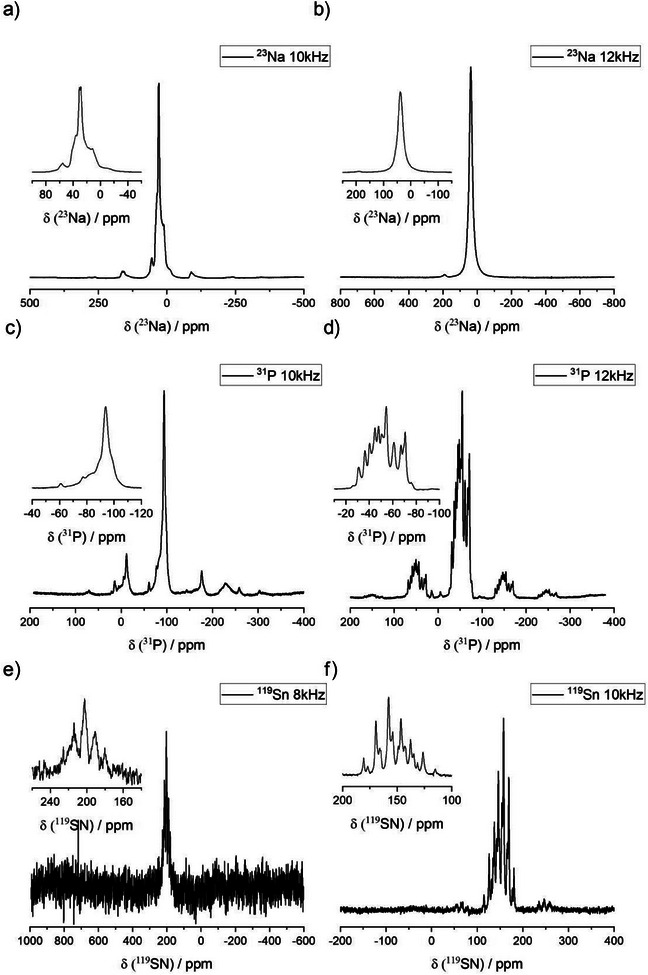
a) ^23^Na, c) ^31^P and e) ^119^Sn MAS–NMR of LT‐Na_8_SnP_4_ as well as b) ^23^Na, d) ^31^P, and f) ^119^Sn MAS–NMR of HT‐Na_8_SnP_4_.

The crystallographic data are supported by ^23^Na, ^31^P, and ^119^Sn solid‐state MAS–NMR measurements. NMR spectra of both phases are given in Figure 6 and additional data on the deconvolution is given in the Supporting Information.

**Figure 7:**
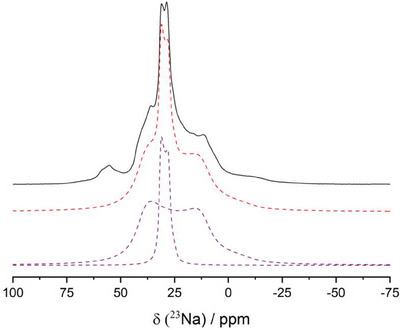
Deconvolution of ^23^Na MAS–NMR of LT‐Na_8_SnP_4_. The observed signal can be distinguished into two separate signals matching the two crystallographic Na‐Positions. Black line: experimental spectrum; Red dashed line: simulated spectrum; Magenta dashed lines: individual contributions from the two Na sites: narrow line centered: Na1: *δ*_iso_ = 32 ppm; *C*_Q_ = 0.9 MHz *η*_Q_ = 0; broader line: Na2: *δ*_iso_ = 48 ppm, *C*_Q _= 2.4 MHz; *η*_Q_ = 0.

The ^23^Na MAS–NMR spectrum of LT‐Na_8_SnP_4_ is shown in Figure 6a and is characterized by two main signals with a minor addition of a side peak at 56 ppm, which we ascribe to an impurity. The main signals at chemical shift values of 32 ppm and 48 ppm are dominated by the quadrupole interaction of the I = 3/2 nucleus ^23^Na. The deconvolution (Figure 7) yields a quadrupolar coupling constant (*C*_Q_) of 0.9 MHz and 2.4 MHz, respectively, for the signal at 32 ppm and 48 ppm and vanishing asymmetry parameters. The integrated areas of the two main signals exhibit a ratio of 1:3 which matches the crystallographic multiplicity of the Na positions at the Wyckoff sites 16c and 48f. The observed quadrupolar parameters are in nice agreement with the results of a quantum chemical calculation using the software package WIEN2k^[^ ^35^, ^36^ ^]^ (Table S9, Supporting Information). Figure 6b shows the ^23^Na MAS–NMR spectrum of HT‐Na_8_SnP_4_ which exhibits one single resonance at a chemical shift of 38 ppm. This indicates ion mobility in the system and matches well with the observation of one distinct signal reported for lithium phosphidotetrelates featuring high Li^+^ mobility.^[^ ^21^, ^22^ ^]^

The ^31^P NMR spectrum of the LT‐phase (Figure 6c) shows one broadened signal at a chemical shift of ‐93.9 ppm which is in agreement with the crystallographic data exhibiting only one P‐position. The broadening of the signal can be attributed to coupling to tin, which is most likely not resolved as there are several NMR active Sn isotopes that will each cause only slightly different coupling. The ^31^P NMR spectrum of the HT‐phase (Figure 6d) exhibits a sharp multiplet of up to thirteen signals with chemical shifts between  −26 and −76 ppm. Regarding the crystallographic data, four signals, one for each phosphorous atom, are expected. However as aforementioned P‐Sn coupling can be very complex due to several NMR active Sn isotopes, the signals can split into different multiplets for each P‐position. Together with the very similar chemical shifts of the signals originating from similar chemical environments of each P‐atoms, this causes an excessive peak overlap that cannot be separated to give precise information on the single P‐atoms. Comparison of the chemical shift observed for both polymorphs however suggest a very similar Sn─P bond situation for both compounds, which is in good agreement with the crystallographic data. Previously reported chemical shifts for resonances in ^31^P MAS–NMR experiments on materials like ZnSnP_2_ or CdSnP_2_ are also in very good agreement with the resonances observed here.^[^ ^37^ ^]^

**Figure 8:**
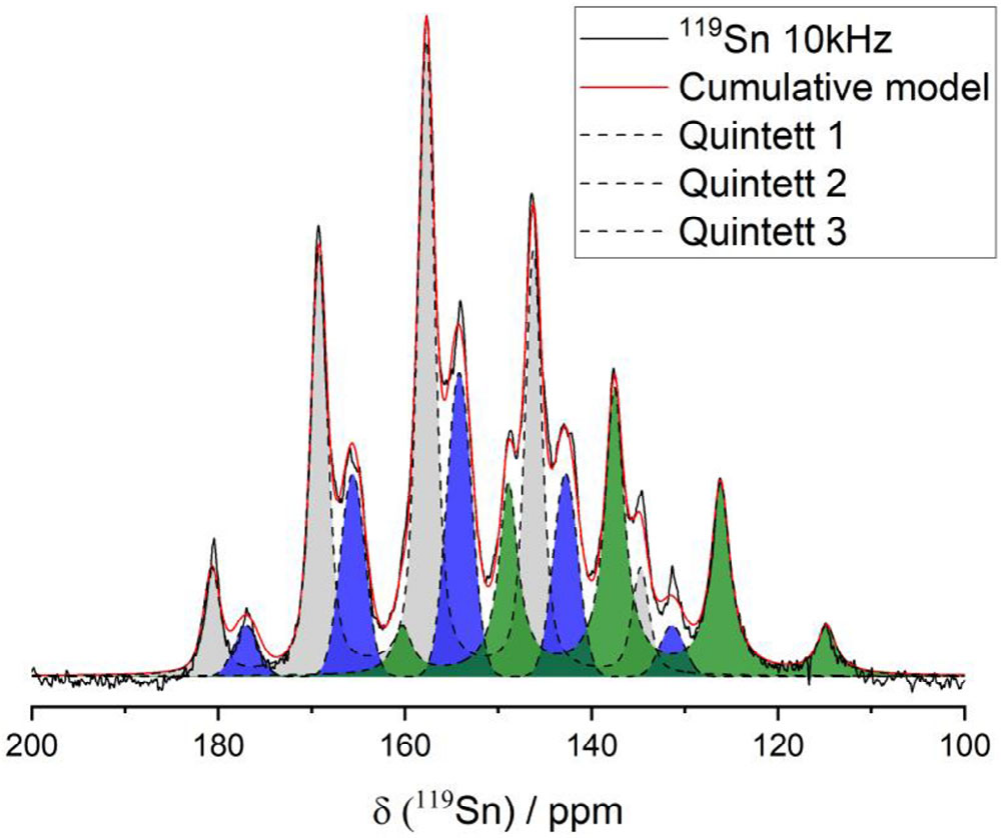
Deconvolution of ^119^Sn MAS–NMR of HT‐Na_8_SnP_4_. Three quintets can be distinguished matching the three crystallographic Sn‐Positions.

The ^119^Sn NMR spectrum of the LT‐phase (Figure 6e) exhibits a single resonance at a chemical shift of 202 ppm, split into a quintett due to the J‐coupling to the four, symmetry equivalent P‐atoms in tetrahedral coordination. A high background noise prevents a more detailed analysis. The ^119^Sn NMR spectrum of the HT‐phase (Figure 6f) exhibits strongly overlapping signals, rendering a deconvolution (Figure 8) crucial for further investigation and a correct assignment of the resonances. The multiplet observed in the spectrum in the range between 114 and 180 ppm can be deconvoluted into three quintets. The three quintets have a chemical shift of 137.5, 154.2, and 157.7 ppm and J_Sn|P_ coupling constants of 1273 Hz, 1278 Hz, and 1286 Hz, respectively, and can be assigned to the three crystallographic Sn‐positions surrounded by four symmetrically equivalent P‐atoms each causing the splitting into a quintet signal. The integrated intensities of the three fits show a ratio of roughly 2:2:4 which is in good agreement with the multiplicity observed for Wyckoff sites 2b, 2e, and 4i occupied by Sn3, Sn1, and Sn2, respectively. The chemical shifts of the Sn atoms as well as the determined coupling constants are comparable to other literature known compounds exhibiting [SnP_4_] tetrahedral units as for example the polymorphs of Li_8_SnP_4_^[^ ^21^ ^]^ exhibiting resonance shifts of 103 to 122 ppm and J_Sn−P_ coupling in the range of 1234–1343 Hz or related compounds with heavier cations like ZnSnP_2_ or CdSnP_2_ exhibiting chemical shift for Sn of 45 and 125 ppm, respectively.^[^ ^37^ ^]^ The downfield shift of the signals for the HT‐Na_8_SnP_4_ polymorph most likely originates from longer Sn─P distances of up to 2.93(2) Å that causes lesser shielding effect of the phosphorous atoms.

### Temperature Dependent Static ^23^Na‐NMR Spectroscopy

The dynamic behavior of the sodium ions in the polymorphs of Na_8_SnP_4_ was evaluated via the temperature‐dependent evolution of the static ^23^Na‐NMR line width within the relevant temperature range. The line width of the ^23^Na central transition is broadened via the homo‐ and heteronuclear dipolar interactions, both of which scale with the second Legendrian (3cos^2^β‐1). Therefore, any dynamic process will cause a (partial) averaging of the orientational dependences and hence induce a narrowing of the ^23^Na NMR central transition.

In the spectra for the LT phase (Figure S10; Supporting Information) no change of the line shape with temperature is visible, suggesting the absence of motion within the temperature range of 180 K – 300 K. All spectra clearly show the presence of two signals with quadrupole parameters of 0.9 MHz and 2.8 MHz and a relative area of 1:3, thus supporting the results from MAS– NMR.

**Figure 9:**
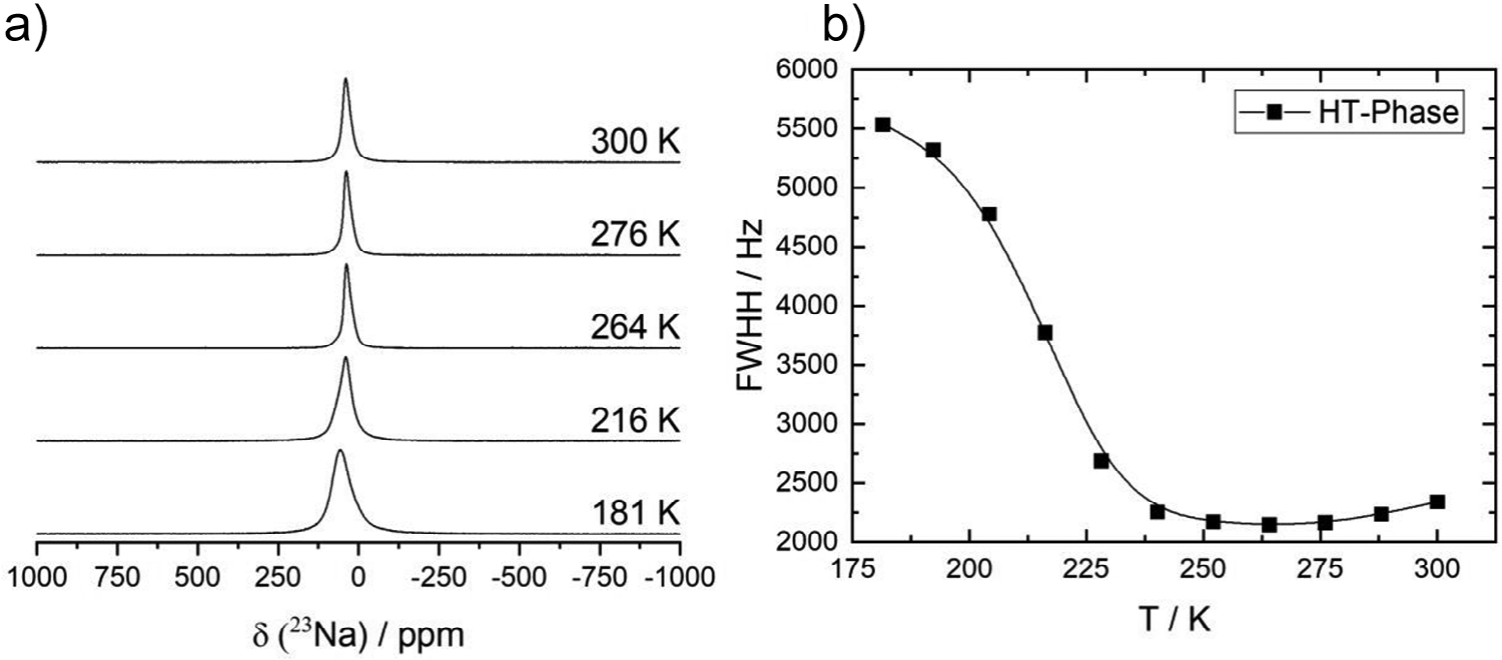
a) Temperature‐dependent evolution of the ^23^Na line shape of HT‐Na_8_SnP_4_ recorded in the temperature range from 181 to 300 K. b) Temperature‐dependent linewidth of the static ^23^Na single pulse excitation measurement (full width at half height) recorded in the temperature range from 181 to 300 K.

For HT‐Na_8_SnP_4_, on the other hand, the spectra (Figure 9a) show a clear line narrowing with increasing temperature from ca 5500 Hz at 175 K down to ≈ 2200 Hz at temperatures above 250 K. Since no individual Na‐positions can be resolved we took the total linewidth of the ^23^Na central transition.

A rough estimation of the activation energy is possible by applying the empirical Waugh–Fedin relation with $E_{A}^{NMR}=0.156\frac{kJ}{mol\;K}\cdot T_{onset}$. The onset temperature here is defined by the temperature at which the line width is reduced to 50% of the difference between the line width of the rigid lattice regime at low temperatures and the motional narrowing regime at high temperature. From this a onset temperature *T*_onset _= 215K can be estimated, translating to an activation energy of *E_A_* = 34 kJ mol^−1^.

### Investigation of the Electrochemical Properties of LT‐Na_8_SnP_4_ and HT‐Na_8_SnP_4_

**Figure 10:**
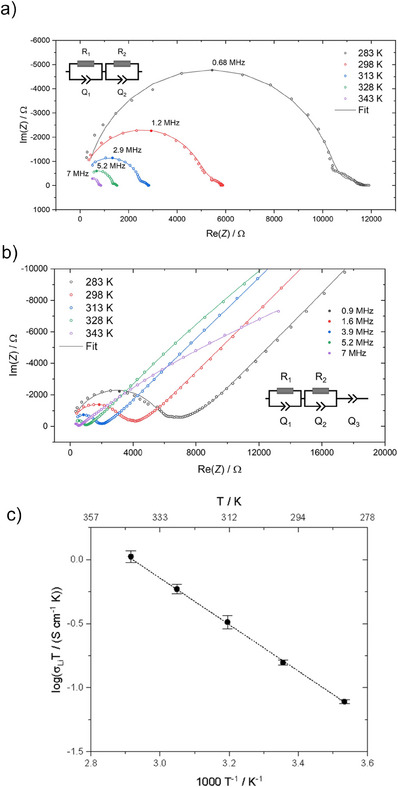
Nyquist plot of the impedance data of LT‐Na_8_SnP_4_ a) and HT‐Na_8_SnP_4_ b) measured under ion blocking conditions, with spectra recorded at temperatures between 283 K and 343 K during a heating cycle. Color coding shows the temperature profile of a cycle for these temperature dependent measurements. Apex frequencies are highlighted with filled dots The equivalent circuits used for the fit are displayed in the corners of the diagrams. Arrhenius plot showing the change of σ_Li_ T of HT‐Na_8_SnP_4_ c) as a function of the inverse temperature with error bars based on the standard deviation from three independent measurements.

To characterize electrochemical properties of the two polymorphs, Na^+^ Ion mobility, activation energies as well as electric conductivities were investigated. Figure 10a,b show impedance spectroscopy measurements under ion blocking conditions at temperatures 283 K, 298 K, 313 K, 328 K, and 343 K. The Nyquist plot of LT‐Na_8_SnP_4_ exhibits a high‐frequency semicircle and a small semicircle in the low frequency regime while missing a polarization tail. The data are interpreted using a model of a series of two parallel arrangements of a resistor (R)and a constant phase element (Q). This impedance response is interpreted as mixed ion‐conducting behavior with significant ionic as well as electronic transport properties.^[^ ^38^ ^]^ However, the static‐NMR measurements reveal that there is no ionic movement, therefore we assign the impedance response to the electronic conductivity of LT‐Na_8_SnP_4_ and the side phase Na_10_Sn_2_P_6_. In contrast features the Nyquist plot of HT‐Na_8_SnP_4_ a high‐frequency semicircle together with a low‐frequency tail corresponding to the electrode polarization. A model using a series of two R/Q and one Q element allows for the first R/Q an attribution of the high‐frequency semicircle to the total ionic conductivity as the sum of both the grain and intergrain ionic conduction, which is not further resolved. The second R/Q corresponds to sample‐electrode phenomena (details see Supporting Information).^[^ ^39^, ^40^ ^]^ The total ionic conductivity of HT‐Na_8_SnP_4_ is determined to 5.3(2) x 10^−4^ S cm^1^ at 298 K. DC polarization measurements in the range from 50–150 mV further reveal electronic conductivities of 6.5(2) x 10^−5^ S cm^−1^ for LT‐Na_8_SnP_4_ and 5(2) x 10^−7^ S cm^−1^ for HT‐Na_8_SnP_4_ at 298 K (Figure S11). The detailed values of the equivalent circuit fits are listed in the Supporting Information (Tables S1,S2). The activation energy for ion transport (Figure 10c) was investigated by temperature‐dependent impedance measurements in the range of 283–‐343 K. Since no ion conduction is present in LT‐Na_8_SnP_4_, only HT‐Na_8_SnP_4_ is investigated, yielding an *E_A_* = 36.1(6) kJ mol^−1^ determined over three independent measurements. The activation energy for HT‐Na_8_SnP_4_ is in good agreement with the value obtained via static NMR measurements.

Comparing the title compounds with Na_8_GeP_4_, the data confirms that the isotypic low temperature modification of Na_8_SnP_4_ exhibits similarly only electronic conductivity, however substitution of Ge into Sn lowered the value by two orders of magnitude. Interestingly, HT‐Na_8_SnP_4_ exhibits a large number of partially occupied sites in combination with ionic conductivity, a similar phenomenon as it is observed in between the α‐ and β‐phases of Li_8_ *Tt*P_4_ (*Tt *= Ge, Sn)^[^ ^21^, ^22^ ^]^ where the β‐phases also exhibit higher ionic conductivity combined with more partially occupied sites for lithium.

## Summary and Discussion

In 1992 Na_3_PS_4_ has been reported as a structurally simple pristine material that exhibited a good Na ion conductivity 0.2 mS cm^−1^ comprising tetrahedral PS_4_ units. Over the years, a step‐by‐step improvement of the conductivity has been achieved by modification of pristine Na_3_PS_4_ with GeS_4_ and WS_4_ building blocks resulting in compositions Na_10_GeP_2_S_12_ and Na_2.9_Sb_0.9_W_0.1_S_4_ reaching values up to 41 mS cm^−1^. Here we report on a novel phosphide‐based compounds Na_8_SnP_4_ with similarly tetrahedral building blocks (SnP_4_ vs. PS_4_) that exhibit as a pristine compound a Na ion conductivity of 0.53 mS cm^−1^ at room temperature, thus surpassing Na_3_PS_4_ by a factor of 2. Na_8_SnP_4_ forms two polymorphs which both be simply synthesized by ball milling the elements and a subsequent annealing procedure. The high temperature modification can be stabilized by rapid cooling. The polymorphs crystallize in the space groups *Fd* $\bar{3}$ *m* and *P* $\bar{4}2$ *c* for the low and high temperature modification, respectively, wherein LT‐Na_8_SnP_4_ is isotypic to Na_8_GeP_4_, while HT‐Na_8_SnP_4_ crystallizes in a new structure type which is closely related to β‐Li_8_SnP_4_.^[^ ^21^ ^]^ In LT‐Na_8_SnP_4_ form the P atoms a cubic closed atom arrangement and all Na atoms are fully ordered and located in either fully occupy tetrahedral and octahedral voids besides empty ones. LT‐Na_8_SnP_4_ shows a very low conductivity which is traced back to an electronic conductivity of 6.5(2) x 10^−5^ S cm^−1^ at 298K. In contrast are the Na atoms of the high temperature modification HT‐Na_8_SnP_4_ distributed over all possible tetrahedral and octahedral voids, which are consequently only partially occupied. According to impedance spectroscopy HT‐Na_8_SnP_4_ exhibits high sodium ion conductivity of 0.53 mS cm^−1^ and low electronic conductivity connected with a low activation energy of *E_A_* = 36.1(6) kJ mol^−1^. The latter is confirmed by static ^23^Na NMR measurements revealing an activation energy of $E_{A}^{NMR}$ = 34 kJ mol^−1^. The ionic conductivity for HT‐Na_8_SnP_4_ is also higher than those reported for other sodium phosphidosilicates with values between 1.76 x 10^−9^ mS cm^−1^ and 0.4 mS cm^−1^.^[^ ^28^ ^]^ In contrast to these known phosphidosilicates that form complex three‐dimensional framework structures, Na_8_SnP_4_ represents a simple structure type with isolated SnP_4_ units and which is based on a *ccp* of P atoms.

The comparison of the properties of the polymorphs show that fully empty sites are not involved in the ion conduction process, but that partially occupied sites are a necessity for sodium ion motion in these compounds. Further, many partially filled Na‐sites as they occur in the HT phase lead to an overall energy landscape flattening. Such effects have also been discussed for lithium argyrodites.^[^ ^34^ ^]^ We further observe a highly distorted octahedral site occupied by Na, which is strongly shifted to the triangular plane of the octahedron shared by a neighboring tetrahedron. A similar effect has been observed in Li_9_AlP_4_ and Li_9_GaP_4_^[^ ^25^ ^]^ and also in sulfide‐based ion conductors.^[^ ^34^ ^]^

Na_8_SnP_4_ presented here is the first phosphide‐based ion‐conducting material in which structural principles similar to those of sulfides occur. It has be shown that the partial substitution of the [PS_4_]^3−^ unit in pristine Na_3_PS_4_ by GeS_4_^4−^ and by SbS_4_^3−^/WS_4_^2−^, as realized in Na_10_GeP_2_S_12_ and Na_2.9_Sb_0.9_W_0.1_S_4_, respectively, leads to an increase in ionic conductivity by more than two orders of magnitude. Thus, the ionic and electronic conductivity can be influenced by isovalent and aliovalent substitution of pristine materials. Na_8_SnP_4_ similarly contains the tetrahedral building block [PS_4_]^3−^ and shows as pristine compound approximately twice the value of ion conductivity of Na_3_PS_4_, indicating that Na_8_SnP_4_ has a great potential for modification and property optimization. Therefore, future investigations will focus on the synthesis of sodium‐containing compounds with possible higher ionic conductivity by isovalent and aliovalent substitution in pristine Na_8_SnP_4_.

## Supporting Information

The Supporting information contains experimental details on the synthesis, powder‐XRD, MAS, static ^29^Na‐NMR measurements, DSC, and impedance measurement procedures. Data concerning MEM analysis, additional crystallographic data, and information on the symmetry relationship of Na_8_SnP_4_.

## Conflict of Interests

The authors declare no conflict of interest.

## Supporting information



Supporting Information

Supporting Information

## Data Availability

The data that support the findings of this study are available in the supplementary material of this article.
