# Supplementary material for: Fast Sodium Ion Conductivity in Pristine Na8SnP4: Synthesis, Structure and Properties of the Two Polymorphs LT‐Na8SnP4 and HT‐Na8SnP4
Source: Angew Chem Int Ed Engl. 2025 Apr 29;64(21):e202419381. doi: 10.1002/anie.202419381 (PMC12087866; doi:10.1002/anie.202419381)
Supplement: Supplementary file 1 — Supporting Information [file ANIE-64-e202419381-s002.docx]

# Fast Sodium Ion Conductivity in Pristine Na_8_SnP_4_ – Synthesis, Structure and Properties of the two Polymorphs LT- Na_8_SnP_4_ and HT-Na_8_SnP_4_

Supporting Information

Content: page

Experimental section S-1

DSC measurement S-6

Rietveld Analysis S-7

Additional crystallographic data S-8

Symmetry relation and Bärnighausen tree S-20

Additional MEM data S-21

Additional NMR data S-23

DC-Polarization curves S-24

Experimental Section

All steps of synthesis and sample preparation were carried out inside an argon-filled glove box (MBraun, *p*(H_2_O), *p*(O_2_) < 0,1 ppm) or in under Ar atmosphere and vacuum (< 2 × 10^−2^ mbar) sealed containers. Prior to use, sodium (Na, rods, Merck-Schuchardt, > 99 %) was cleaned from oxide layers. Tin (Sn, powder, Alfa Aesar, 99,8%) and red phosphorus (P, powder, Sigma-Aldrich, 97 %) were used without any further purification. All obtained compounds are sensitive to oxygen and moisture with the latter showing vigorous reaction that results in flammable and toxic gases. Therefore, the disposal must be addressed under proper ventilation and in small amounts at a time.

**Synthesis of LT-Na_8_SnP_4_ and HT-Na_8_SnP_4_.**

LT-Na_8_SnP_4_ was synthesized in a two-step synthesis from the elements via ball milling and subsequent annealing. Batches of the “reactive mixture” with *m* = 3.0 g containing sodium (1293,6 mg, 56.29 mmol, 8.0 equiv.), tin (834.9 mg, 7.03 mmol, 1.0 equiv.) and red phosphorus (898.4 mg, 29.0 mmol, 4.0 equiv.) were prepared by mechanochemical milling (Retch PM 100 planetary mill, 18 h, 350 rpm, intervals of 10 min with direction reversal and subsequent 5 min resting) using a tungsten carbide milling set (50 mL jar with 3 balls with a diameter of 15 mm each).

The obtained black reactive mixture was sealed into niobium crucibles in batches of 200mg using an arc furnace (Edmund Bühler MAM1). The sealed ampules were enclosed in evacuated silica reaction containers. The containers were heated in a tube furnace (HTM Reetz Loba) with 4 K·min^‑1^ up to 873 K, dwelled for 12 h and cooled down at a rate of 2 K·min^‑1^. After grinding, a black powder was obtained.

Analogously to LT-Na_8_SnP_4_, HT-Na_8_SnP_4_ was synthesized using the procedure as described above followed by quenching of the hot reaction crucibles in an ice/water bath after annealing instead of slow cooling.

**Powder X-ray diffraction.**

For powder X-ray diffraction (PXRD) measurements, the samples were ground in an agate mortar and sealed inside 0.3 mm glass capillaries. PXRD measurements were performed at room temperature on a STOE Stadi P diffractometer (Ge(111) monochromator, Cu *Kα*_1_ radiation, *λ* = 1.54056 Å) with a Dectris MYTHEN 1K detector in Debye-Scherrer geometry. The raw powder data were processed with the software package WinXPOW.^[1]^

**Synchrotron X-ray data and Rietveld refinement.**

The samples for the powder X-ray synchrotron diffraction measurements were filled in capillaries with a diameter of 0.3 mm. PXRD measurements for solving the structure of LT-Na_8_SnP_4_ were performed on the P02.1 beamline^[2]^ at the PETRA III Synchrotron (DESY, Hamburg, Germany). The data was collected using Varex XRD 4343CT detector with 150 µm x 150 µm pixel size. The distance between detector and sample was fixed to 1.503 m. The energy of the synchrotron radiation was set at 60 keV (λ = 0.20707 Å). The measurements used for solving the structure of HT-Na_8_SnP_4_ were measured at the Swiss Norwegian Beamline^[3]^ (ESRF, Grenoble, France) with data was collected using 2M PILATUS3 detector with 172 µm x 172 µm pixel size. The distance between detector and sample was varied in three steps between 0.13912 m and 0.69312 m. The energy of the synchrotron radiation was set at 17,84 keV (λ = 0.69483 Å). In both experimental setups the reference NIST SRM 660a LaB_6_ is used as a standard powder for the calibration of the diffraction data.

The temperature dependent powder X-ray Synchrotron diffraction measurements were performed at the P02.1 beamline. The experimental setup was similar to this on LT-Na_8_SnP_4_ Data collection was performed under stepwise heating using hot air blower system from FMB Oxford: at temperatures below 550°C the data collection was carried out every 10 °C, whilst at higher temperatures (below 750°C) the temperature increment was reduced to 5°C.

Rietveld refinements of LT-Na_8_SnP_4_ and HT-Na_8_SnP_4_ were executed using the full profile Rietveld method within the FullProf program package.^[4]^ The Thomson-Cox-Hastings^[5]^ profile function was used to model the peak profile shape. Background contribution was determined using a linear interpolation between selected data points in non-overlapping regions. Scale factor, profile shape parameters, resolution (Caglioti) parameters and lattice parameters were refined without restrictions. For LT-Na_8_SnP_4_ the structure model of Na_8_SnSb_4_ was used as an input. All atom displacement parameters were refined without restrictions. Refinement of the site occupancy factors (S.O.F) for the Na positions resulted in 0.997 for Na1 and 0.989 for Na2. Thus the occupancy within experimental errors were set to 1 in the final refinement which is also in agreement for a charge balanced compound Na_8_SnP_4_. For HT-Na_8_SnP_4_, the structure was found well simulated by the translationengleiche symmetry lowering from *β*‑Li_8_SnP_4_. Herein, a combination of two measurements at distinct sample-to-detector distances of 0.49312 m and 0.69413 m were refined simultaneously. Due to the lack of high angle data for HT-Na_8_SnP_4_, fractional coordinates of Na13, Na14 and Na15 were fixed to the center of the octahedral site. Here, Displacement parameters within each atom sort (Sn, P, Na) as well as within tetrahedrally coordinated and octahedrally coordinated Na-Sites were constrained, respectively, to be the same and refined. Special attention has to be granted to the four octahedral sites Na13-Na16, where a free refinement of their fractional coordinates resulted in very strong deviation from the octahedral centre and, in the case of Na13-Na15 led to unreliable results. Consequently, the fractional coordinates of Na13, Na14 and Na15 were fixed in the symmetric centre of their respective octahedral site. The restricted Na^+^ ions located at octahedral sites Na13-Na15 exhibit Na-P bond distances ranging from 3.07(1) Å to 3.64(1) Å while the highly distorted Na16 site shows Na-P bond distances ranging from 2.55(5) Å to 4.85(5) Å. The Na-P bond distances for the residual tetrahedrally coordinated sites are in the range of 2.68(2) Å - 3.28(2) Å which greater compared to literature known compounds like Na_8_GeP_4_ (2.97-3.04 Å)^[6]^ and likely originates from the disorder phenomenon observed for the octahedral sites. The formed tetrahedra similarly show different levels of distortion with P-Na-P bond angles reaching from 93.9(3)° to 122.2(3)° which is high compared to other known compounds with tetrahedrally coordinated Na-sites.

Furthermore, Maximum Entropy Method (MEM) based on data from temperature dependent powder X-ray Synchrotron diffraction measurements was conducted. Herein, Analysis of electron densities was carried out using maximum entropy method as implemented in DYSNOMIA software.^[7]^ Structure factors and their phases were modelled using Rietveld refinement and used as input information. 3D distribution of electron densities was calculated on 128x128x128 grid of elementary cell and visualised using VESTA.^[8]^

**Differential scanning calorimetry (DSC).**

For thermal analysis, samples were sealed in a niobium ampoule and measured on a DSC machine (Netzsch, DSC 404 Pegasus) under a constant gas flow of 75 mL∙min^−1^. The sample was heated to 973 K and cooled to 473 K twice at a rate of 5 K∙min^−1^. To determine the onset temperatures of the DSC signals, the PROTEUS Thermal Analysis software^[9]^ was used and visualization was realized using OriginPro 2021.

**NMR spectroscopy**

Nuclear magnetic resonance measurements were used to confirm the determined crystal structure. The MAS-NMR measurements were carried out using a Bruker Avance 300 NMR device operating at a magnetic field strength of 7 T with resonance frequencies of 121.5 MHz, 111.9 MHz, and 79.4 MHz for ^31^P, ^119^Sn and ^23^Na, respectively. MAS NMR spectra were recorded at room temperature following a single excitation pulse using 4mm ZrO_2_ rotors and spinning frequencies of 8 – 12 kHz. Recycle delays were set to 10 seconds (^119^Sn) and 30 seconds (^31^P, ^23^Na) and the spectra referenced to (NH_4_)H_2_PO_3_ at 1.11 ppm (^31^P), SnO_2_ at -604.3 ppm (^119^Sn) and 1M aq. NaCl at 0 ppm (^23^Na). The processing and evaluation of the measured data was performed employing the software MestReNova^[10]^ and DMFit.^[11]^

To the end of evaluating possible sodium mobility static solid state NMR measurements (single-pulse excitation as well as a solid echo sequence(90-τ-90-acq)) were performed. The samples were sealed in glass ampules and were measured under a constant nitrogen flow. The temperature calibration was performed employing the 207Pb NMR resonance of Pb(NO_3_)_3_ as a chemical shift thermometer.^[12]^ Recycle delays of 1 – 30 s were used depending on the temperature. Processing of the data was performed using the software packages TopSpin and DMFit.^[11]^

Impedance Spectroscopy and DC Conductivity Measurements

The ionic conductivity of LT- and HT-Na_8_SnP_4_ are determined by impedance spectroscopy in a commercial cell with tungsten carbide electrodes (RHD instruments, CompreCell). Powdered samples (250 mg) were compressed in a hydraulic press with a pressure of 400 MPa to at least 88% of the crystal density. During electrochemical measurements, a constant pressure of 150 MPa was applied on the cell by the compression of springs. Impedance spectra were recorded on a Bio-Logic potentiostat (VMP-300) in a frequency range from 7 MHz to 100 mHz at a potentiostatic excitation of ±50 mV. The measurement was performed on VMP 300 potentiostat (*BioLogic*). Data were treated using the software RelaxIS (V 3.0.18.15). The measurements were performed in a climate cabinet (ESPEC, LU-114). For the determination of the activation energy of the sodium ion conduction, the cell temperature was set to 283, 298, 313, 328, and 343 K. Prior to EIS measurements, the cell rested 150 min to allow for thermal equilibration. The electronic conductivity was determined with the same setup using a potentiostatic polarization applying voltages of 50, 100, and 150 mV for 8 h each.

The Nyquist plot of HT-Na_8_SnP_4_ features a high-frequency semicircle together with a low-frequency tail corresponding to the electrode polarization. These can be modelled by a series of two parallel arrangements of a resistor and a constant phase element (R/Q) and one Q element. The high-frequency semicircle can be attributed to the total ionic conductivity as the sum of both the grain and intergrain ionic conduction for HT-Na_8_SnP_4_, which could not be further resolved and corresponds to the first (R/Q). For this constant phase element, the fit of the data at 298 K resulted in α-values of 0.91(2) as well as Q-values of 1.0(1) x 10^-10^ F s^(α-1)^ for HT-Na_8_SnP_4_. The total ionic conductivity of HT‑Na_8_SnP_4_ was determined to 5.3(2) x 10^-4^ S cm^1^ at 298 K. The second (R/Q) cannot be assigned clearly. Since its Q- and α-values are in the range of 10^‑6^ F s^(α-1)^ and 0.4, respectively, which is several orders of magnitude above bulk and grain boundary processes, we attribute it to sample-electrode phenomena.

**Table S1:** Fitting parameters LT-Na_8_SnP_4_.

| T / K | R_1_ / Ω | Q_1_ / F s^(α‑1)^ | α_1_ | R_2_ / Ω | Q_2_ / F s^(α‑1)^ | α_2_ |
| --- | --- | --- | --- | --- | --- | --- |
| 283 | 9629 | 3.7 × 10^-11^ | 0.97 | 2205 | 1.6 × 10^-5^ | 0.29 |
| 298 | 4808 | 5.4 × 10^-11^ | 0.94 | 1054 | 1.6 × 10^-6^ | 0.51 |
| 313 | 2424 | 4.8 × 10^-11^ | 0.95 | 401 | 1.1 × 10^-6^ | 0.63 |
| 328 | 1328 | 5.6 × 10^-11^ | 0.94 | 192 | 3.2 × 10^-6^ | 0.6 |
| 343 | 772 | 9.6 × 10^-11^ | 0.91 | 87 | 6.7 × 10^-6^ | 0.61 |

**Table S2:** Fitting parameters HT-Na_8_SnP_4_.

| T / K | R_1_ / Ω | Q_1_ / F s^(α‑1)^ | α_1_ | R_2_ / Ω | Q_2_ / F s^(α‑1)^ | α_2_ | Q_3_ / F s^(α‑1)^ | α_3_ |
| --- | --- | --- | --- | --- | --- | --- | --- | --- |
| 283 | 5393 | 2.7 × 10^-10^ | 0.86 | 2487 | 4.2 × 10^-6^ | 0.43 | 2.8 × 10^-5^ | 0.51 |
| 298 | 2869 | 1.2 × 10^-10^ | 0.92 | 1746 | 3.5 × 10^-6^ | 0.42 | 3.7 × 10^-5^ | 0.5 |
| 313 | 1437 | 9.9 × 10^-11^ | 0.93 | 910 | 1.5 × 10^-5^ | 0.34 | 4.6 × 10^-5^ | 0.49 |
| 328 | 708 | 1.4 × 10^-10^ | 0.93 | 1.3 × 10^5^ | 1.5 × 10^-7^ | 0.54 | 6.3 × 10^-4^ | 0.13 |
| 343 | 515 | 2.4 × 10^-9^ | 0.74 | 6356 | 1.6 × 10^-4^ | 0.53 | 1.1 × 10^-4^ | 0.37 |

Differential Scanning Calorimetry


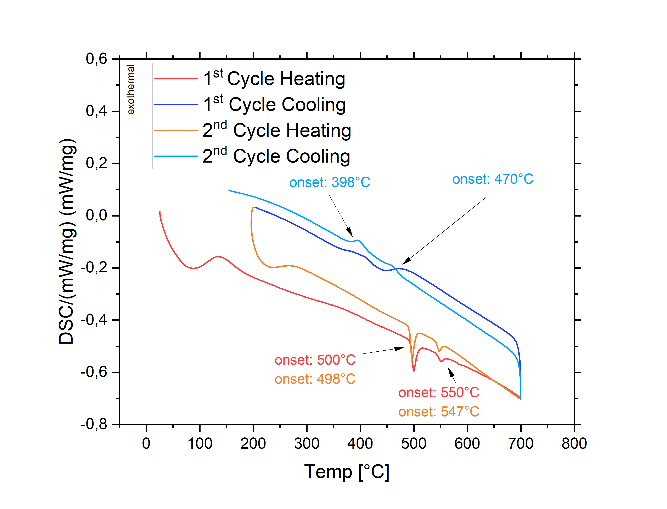

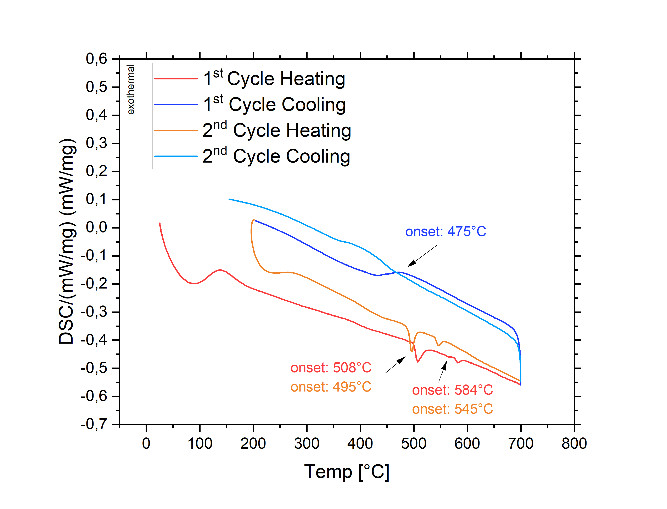


c)

b)

a)


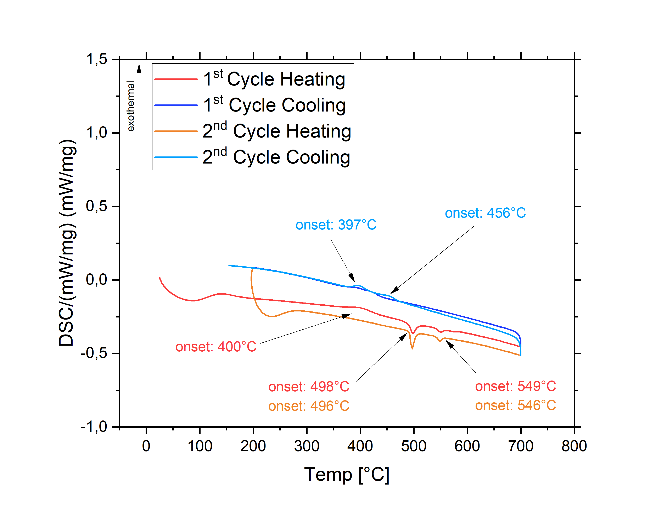


**Figure S1:** Differential scanning calorimetry experiments. a) Measurement of the “reactive mixture” after ball-milling step. b) Measurement of LT-Na_8_SnP_4_. c) Measurement of HT-Na_8_SnP_4_. All measurement were performed in two cycles from 200-700°C. Above 700°C only decomposition occurs.

Rietveld Analysis


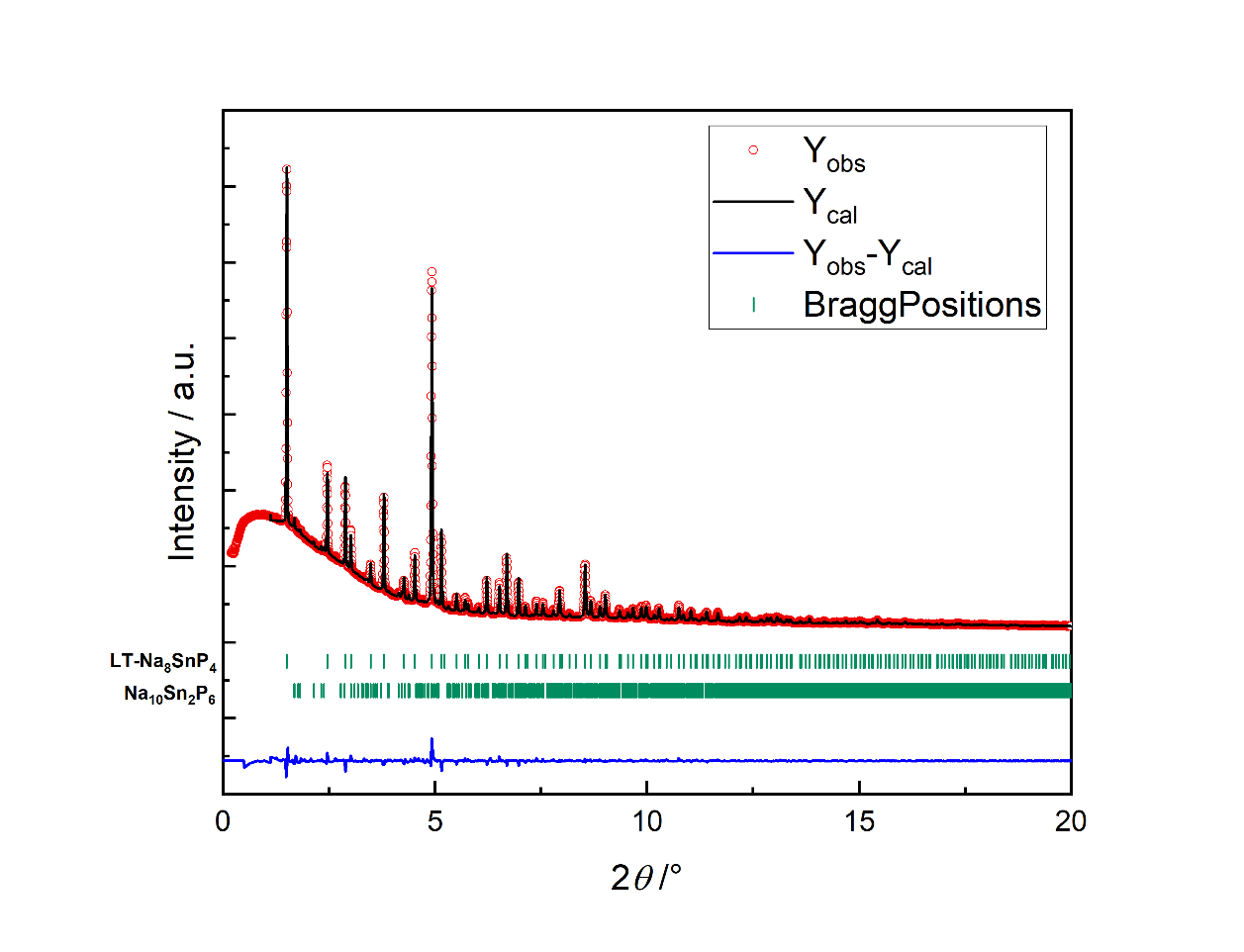




b)

a)

**Figure S2:** Rietveld refinement of the powder synchrotron X-ray data of a) LT- and b) HT-Na_8_SnP_4_. The red line indicates observed intensities, the black line calculated intensities and the blue line shows the difference. Bragg positions are depicted as green dashes. Synchrotron radiation was set at 60 keV (λ = 0.20707 Å) for the measurement of the LT-Phase and 17,84 keV (λ = 0.69483 Å) for the measurement of the HT-Phase. The Na_8_SnP_4_ : Na_10_Sn_2_P_6_ ratio for the LT Phase is 96,67 wt.-%: 3,33 wt.-% and for the HT Phase 96,69 wt.-%: 3,31 wt.-% respectively.

Statement:

The Chi2 – Values of the Refinements were obtained as 3.92 and 31426 for LT- and HT-Na_8_SnP_4_, respectively. It is observed that, due to extremely high counting rates supplied by the brilliant synchrotron radiation, the standard indicators like Rexp and Chi2 become not informative/representative enough). In the Rietveld software (including FullProf) typically two sets of indices are calculated. Per definition

| *R*_exp_  = | { | ( *N* - *P* + *C* ) | / | Σ *i* | *w*_i_ *y*_i_(obs)^2^ | }^1/2^ × 100% |
| --- | --- | --- | --- | --- | --- | --- |

where *N* is the total number of observations, *i*, (as used in the summation), *P* is the number of parameters refined, and *C* is the number of constraints used in the refinement. For most structural refinements with powder diffraction data, the expression (*N* - *P* + *C*) is dominated by the size of *N*. On the other hand the values are scaled by the weighted intensity, i.e. increase of counting rate drives the Rexp to lower values. The ratio of the above two *R*-factors gives the chi-squared value for the fit:

χ^2^ = ( *R*_wp_ / *R*_exp_ )^2^

The usage of large area 2D detectors further adds to the complexity. Typically, this is solved by the division of intensities by the certain factor (e.g. 10000), which we consider not fully professional. However, there is no common and standard solution in the literature.

To avoid confusions, we decided to omit the Chi2 values from the Manuscript.

Crystal Tables

**Table S3:** Atomic coordinates and isotropic atomic displacement parameters of LT-Na_8_SnP_4_ at RT. Tetrahedral positions are marked in blue and octahedral positions in red, respectively.

| **Atom** | **Wyckoff positions** | **S.O.F.** | ***x*/*a*** | ***y*/*b*** | ***z*/*c*** | ***U*_iso_ [Å^2^]** |  |
| --- | --- | --- | --- | --- | --- | --- | --- |
| P1 | 32e | 1 | 0.23471(6) | 0.23471(6) | 0.23471(6) | 0.0082(5) |  |
| Sn1 | 8a | 1 | 1/8 | 1/8 | 1/8 | 0.0148(2) |  |
| Na1 | 16c | 1 | 0 | 0 | 0 | 0.034(1) |  |
| Na2 | 48f | 1 | 0.4013(1) | 1/8 | 1/8 | 0.0165(6) |  |

**Table S4:** Atomic coordinates and isotropic atomic displacement parameters of HT-Na_8_SnP_4_ at RT. Tetrahedral positions are marked in blue and octahedral positions in red, respectively.

| **Atom** | **Wyckoff positions** | **S.O.F.** | ***x*/*a*** | ***y*/*b*** | ***z*/*c*** | ***U*_iso_ [Å^2^]** |  |
| --- | --- | --- | --- | --- | --- | --- | --- |
| Sn1 | 2e | 1 | 0 | 0 | 0 | 0.0021(5) |  |
| Sn2 | 4i | 1 | 0.7487(4) | 1/2 | 1/4 | 0.0021(5) |  |
| Sn3 | 2b | 1 | 0 | 1/2 | 3/4 | 0.0021(5) |  |
| P1 | 8n | 1 | 0.611(1) | 0.353(1) | 0.8789(6) | 0.020(1) |  |
| P2 | 8n | 1 | 0.888(1) | 0.389(1) | 0.6408(8) | 0.020(1) |  |
| P3 | 8n | 1 | 0.856(1) | 0.379(1) | 0.1248(8) | 0.020(1) |  |
| P4 | 8n | 1 | 0.105(1) | 0.120(1) | 0.1019(7) | 0.020(1)) |  |
| Na1 | 2f | 1 | 1/2 | 1/2 | 0 | 0.024(1) |  |
| Na2 | 2a | 1 | 0 | 0 | 1/4 | 0.024(1)) |  |
| Na3 | 2d | 1 | 1/2 | 0 | 3/4 | 0.024(1) |  |
| Na4 | 2c | 1 | 1/2 | 1/2 | 1/4 | 0.024(1) |  |
| Na5 | 4h | 1 | 0.7467(9) | 1/2 | 3/4 | 0.024(1) |  |
| Na6 | 4j | 1 | 0 | 1/4 | 1/4 | 0.024(1) |  |
| Na7 | 4m | 1 | 1/2 | 0 | 0.9959(8) | 0.024(1) |  |
| Na8 | 4g | 1 | 0 | 0.248(2) | 3/4 | 0.024(1) |  |
| Na9 | 8n | 0.78(1) | 0.265(1) | 0.971(1) | 0.4914(9) | 0.024(1) |  |
| Na10 | 8n | 1 | 0.766(1) | 0.250(1) | 0.7811(8) | 0.024(1) |  |
| Na11 | 8n | 0.57(2) | 0.239(2) | 0.275(1) | 0.999(1) | 0.024(1) |  |
| Na12 | 8n | 0.64(1) | 0.769(1) | 0.491(2) | 0.517(1) | 0.024(1) |  |
| Na13 | 8n | 0.21(1) | 0.87500 | 0.37500 | 0.87500 | 0.21(1) |  |
| Na14 | 8n | 0.62(3) | 0.62500 | 0.37500 | 0.12500 | 0.21(1) |  |
| Na15 | 8n | 0.70(5) | 0.62500 | 0.12500 | 0.87500 | 0.21(1) |  |
| Na16 | 8n | 0.43(2) | 0.796(5) | 0.167(5) | 0.056(4) | 0.21(1) |  |

**Table S5:** Selected atomic distances up to 4Å of LT-Na_8_SnP_4_ at RT.

| **Atom pair** | | | *d* 1,2 [Å] |
| --- | --- | --- | --- |
| P1 | Sn1 | 1x | 2.5886(8) |
|  | Na2 | 3x | 2.931(1) |
|  | Na2 | 3x | 3.101(1) |
|  | Na1 | 3x | 3.2109(8) |
| Sn1 | P1 | 4x | 2.5886(8) |
|  | Na1 | 4x | 2.9493(0) |
|  | Na2 | 6x | 3.7648(0) |
| Na1 | Sn1 | 2x | 2.9493(0) |
|  | Na2 | 6x | 3.1703(8) |
|  | P1 | 6x | 3.2109(8) |
| Na2 | P1 | 2x | 2.931(1) |
|  | P1 | 2x | 3.101(1) |
|  | Na1 | 2x | 3.170(1) |
|  | Na2 | 4x | 3.4433(2) |
|  | Sn1 | 1x | 3.764(1) |

**Table S6:** Bond angles of up to 4Å of SnP_4_ and NaP_4_ tetrahedra and NaP_6_ octahedra in LT-Na_8_SnP_4_ at RT.

| **Atom 1 – 2 - 3** | | | Angle [°] | **Atom 1 – 2 - 3** | | | Angle [°] |
| --- | --- | --- | --- | --- | --- | --- | --- |
| P1 | Sn1 | P1 | 109.47(2) | P1 | Na1 | P1 | 82.33(2) |
| P1 | Sn1 | P1 | 109.47(2) | P1 | Na1 | P1 | 82.33(2) |
| P1 | Sn1 | P1 | 109.47(2) | P1 | Na1 | P1 | 97.66(2) |
| P1 | Sn1 | P1 | 109.47(2) | P1 | Na1 | P1 | 180.00(2) |
| P1 | Sn1 | P1 | 109.47(2) | P1 | Na1 | P1 | 97.66(2) |
| P1 | Sn1 | P1 | 109.47(2) | P1 | Na1 | P1 | 97.66(2) |
| P1 | Na1 | P1 | 97.66(2) | P1 | Na1 | P1 | 82.33(2) |
| P1 | Na1 | P1 | 82.33(2) | P1 | Na2 | P1 | 134.42(2) |
| P1 | Na1 | P1 | 82.33(2) | P1 | Na2 | P1 | 106.46(2) |
| P1 | Na1 | P1 | 180.00(2) | P1 | Na2 | P1 | 106.46(2) |
| P1 | Na1 | P1 | 97.66(2) | P1 | Na2 | P1 | 106.46(2) |
| P1 | Na1 | P1 | 97.66(2) | P1 | Na2 | P1 | 106.46(2) |
| P1 | Na1 | P1 | 180.00(2) | P1 | Na2 | P1 | 85.90(2) |
| P1 | Na1 | P1 | 82.33(2) |  |  |  |  |

**Table S7:** Selected atomic distances up to 4Å of HT-Na_8_SnP_4_ at RT.

| **Atom pair** | | | *d* 1,2 [Å] | **Atom pair** | | | *d* 1,2 [Å] |
| --- | --- | --- | --- | --- | --- | --- | --- |
| Sn1 | P4 | 4x | 2.55(1) | Na8 | P2 | 2x | 2.83(2) |
|  | Na2 | 1x | 3.3826(0) |  | Na13 | 2x | 2.92(1) |
|  | Na2 | 1x | 3.3831(0) |  | Na10 | 2x | 3.16(1) |
|  | Na9 | 4x | 3.58(2) |  | P4 | 2x | 3.20(2) |
|  | Na16 | 4x | 3.61(6) |  | Na9 | 2x | 3.29(1) |
| Sn2 | P1 | 2x | 2.67(1) |  | Na2 | 1x | 3.32(2) |
|  | P3 | 2x | 2.74(1) |  | Sn3 | 1x | 3.38(2) |
|  | Na14 | 2x | 2.904(3) |  | Na16 | 2x | 3.50(6) |
|  | Na15 | 2x | 2.924(3) | Na9 | Na13 | 1x | 2.51(1) |
|  | Na4 | 1x | 3.338(5) |  | P4 | 1x | 2.77(2) |
|  | Na3 | 1x | 3.373(5) |  | Na16 | 1x | 2.82(6) |
|  | Na10 | 2x | 3.59(1) |  | P3 | 1x | 2.83(2) |
|  | Na12 | 2x | 3.63(1) |  | Na16 | 1x | 2.83(6) |
| Sn3 | P2 | 4x | 2.57(1) |  | P4 | 1x | 3.04(2) |
|  | Na13 | 1x | 2.9139(0) |  | P2 | 1x | 3.08(2) |
|  | Na13 | 3x | 2.9144(0) |  | Na15 | 1x | 3.11(1) |
|  | Na8 | 2x | 3.38(2) |  | Na7 | 1x | 3.17(2) |
|  | Na5 | 2x | 3.38(1) |  | Na8 | 1x | 3.29(1) |
|  | Na7 | 2x | 3.43(1) |  | Na11 | 1x | 3.33(2) |
| P1 | Sn2 | 1x | 2.67(1) |  | Na6 | 1x | 3.52(1) |
|  | Na11 | 1x | 2.71(2) |  | Sn1 | 1x | 3.58(2) |
|  | Na10 | 1x | 2.82(2) |  | Na11 | 1x | 3.59(2) |
|  | Na12 | 1x | 2.85(2) |  | Na10 | 1x | 3.96(2) |
|  | Na1 | 1x | 2.96(1) | Na10 | Na13 | 1x | 2.56(1) |
|  | Na4 | 1x | 3.02(1) |  | P1 | 1x | 2.82(2) |
|  | Na15 | 1x | 3.07(1) |  | Na15 | 1x | 2.83(1) |
|  | Na14 | 1x | 3.19(1) |  | P4 | 1x | 2.93(2) |
|  | Na5 | 1x | 3.20(1) |  | Na11 | 1x | 3.02(2) |
|  | Na12 | 1x | 3.28(2) |  | P2 | 1x | 3.12(1) |
|  | Na14 | 1x | 3.346(8) |  | Na8 | 1x | 3.16(1) |
|  | Na14 | 1x | 3.452(8) |  | P3 | 1x | 3.21(2) |
|  | Na13 | 1x | 3.54(1) |  | Na16 | 1x | 3.23(5) |
|  | Na14 | 1x | 3.64(1) |  | Na14 | 1x | 3.29(1) |
| P2 | Sn3 | 1x | 2.57(1) |  | Na5 | 1x | 3.38(1) |
|  | Na12 | 1x | 2.68(2) |  | Na6 | 1x | 3.39(1) |
|  | Na5 | 1x | 2.82(1) |  | Sn2 | 1x | 3.59(1) |
|  | Na8 | 1x | 2.83(2) |  | Na11 | 1x | 3.81(2) |
|  | Na16 | 1x | 2.83(6) |  | Na16 | 1x | 3.90(5) |
|  | Na7 | 1x | 2.88(1) |  | Na9 | 1x | 3.96(2) |
|  | Na11 | 1x | 2.97(2) | Na11 | Na16 | 1x | 1.55(6) |
|  | Na9 | 1x | 3.08(2) |  | Na15 | 1x | 2.65(2) |
|  | Na10 | 1x | 3.12(1) |  | P1 | 1x | 2.71(2) |
|  | Na13 | 2x | 3.17(1) |  | Na14 | 1x | 2.82(2) |
|  | Na13 | 1x | 3.18(1) |  | Na12 | 1x | 2.91(3) |
|  | Na14 | 1x | 3.54(1) |  | P2 | 1x | 2.97(2) |
|  | Na15 | 1x | 3.60(1) |  | Na10 | 1x | 3.02(2) |
| P3 | Sn2 | 1x | 2.74(1) |  | P4 | 1x | 3.07(2) |
|  | Na9 | 1x | 2.83(2) |  | P3 | 1x | 3.08(2) |
|  | Na12 | 1x | 2.84(2) |  | Na13 | 1x | 3.20(2) |
|  | Na7 | 1x | 3.00(1) |  | Na9 | 1x | 3.33(2) |
|  | Na3 | 1x | 3.03(1) |  | Na9 | 1x | 3.59(3) |
|  | Na11 | 1x | 3.07(2) |  | Na12 | 1x | 3.68(3) |
|  | Na14 | 1x | 3.10(1) |  | Na10 | 1x | 3.81(2) |
|  | Na6 | 1x | 3.10(1) | Na12 | P2 | 1x | 2.68(2) |
|  | Na16 | 1x | 3.10(6) |  | Na13 | 1x | 2.71(2) |
|  | Na10 | 1x | 3.21(2) |  | P3 | 1x | 2.84(2) |
|  | Na15 | 1x | 3.30(1) |  | P1 | 1x | 2.85(2) |
|  | Na13 | 1x | 3.38(1) |  | Na15 | 1x | 2.85(2) |
|  | Na15 | 1x | 3.39(1) |  | Na14 | 1x | 2.88(2) |
|  | Na15 | 1x | 3.61(1) |  | Na11 | 1x | 2.91(3) |
| P4 | Na16 | 1x | 2.55(5) |  | Na7 | 1x | 3.11(2) |
|  | Sn1 | 1x | 2.55(1) |  | Na5 | 1x | 3.15(1) |
|  | Na9 | 1x | 2.77(2) |  | Na14 | 1x | 3.27(2) |
|  | Na10 | 1x | 2.93(1) |  | P1 | 1x | 3.28(2) |
|  | Na2 | 1x | 2.93(1) |  | Na1 | 1x | 3.62(2) |
|  | Na6 | 1x | 3.01(1) |  | Sn2 | 1x | 3.63(1) |
|  | Na9 | 1x | 3.04(2) |  | Na11 | 1x | 3.68(3) |
|  | Na11 | 1x | 3.07(2) |  | Na16 | 1x | 3.98(7) |
|  | Na8 | 1x | 3.20(1) | Na13 | Na9 | 1x | 2.51(1) |
|  | Na15 | 1x | 3.44(1) |  | Na10 | 1x | 2.56(1) |
|  | Na13 | 1x | 3.62(1) |  | Na12 | 1x | 2.71(2) |
| Na1 | Na14 | 4x | 2.9141(0) |  | Sn3 | 1x | 2.9146) |
|  | P1 | 4x | 2.96(1) |  | Na8 | 1x | 2.93(1) |
|  | Na4 | 1x | 3.3826(0) |  | Na5 | 1x | 2.931() |
|  | Na4 | 1x | 3.3831(0) |  | Na7 | 1x | 2.946() |
|  | Na12 | 4x | 3.62(2) |  | P2 | 2x | 3.17(1) |
| Na2 | P4 | 4x | 2.93(1) |  | P2 | 1x | 3.18(1) |
|  | Na8 | 1x | 3.3289(0) |  | Na11 | 1x | 3.20(2) |
|  | Na8 | 1x | 3.3293(0) |  | P3 | 1x | 3.39(1) |
|  | Na6 | 2x | 3.356(0) |  | P1 | 1x | 3.54(1) |
|  | Sn1 | 1x | 3.3826(0) |  | P4 | 1x | 3.62(1) |
|  | Sn1 | 1x | 3.3831(0) |  | Na16 | 1x | 3.86(6) |
| Na3 | Na15 | 4x | 2.9139(0) | Na14 | Na11 | 1x | 2.82(2) |
|  | P3 | 4x | 3.03(1) |  | Na12 | 1x | 2.87(2) |
|  | Na7 | 2x | 3.32(1) |  | Na5 | 1x | 2.897(7) |
|  | Na6 | 2x | 3.356(0) |  | Sn2 | 1x | 2.904(3) |
|  | Sn2 | 1x | 3.3732(0) |  | Na4 | 1x | 2.9141(0) |
|  | Sn2 | 1x | 3.3736(0) |  | Na1 | 1x | 2.9144(0) |
| Na4 | Na14 | 4x | 2.9139(0) |  | P3 | 1x | 3.10(1) |
|  | P1 | 4x | 3.02(1) |  | P1 | 1x | 3.19(1) |
|  | Na5 | 1x | 3.3265(0) |  | Na12 | 1x | 3.27(2) |
|  | Na5 | 1x | 3.3269(0) |  | Na10 | 1x | 3.29(1) |
|  | Sn2 | 1x | 3.338(5) |  | P1 | 1x | 3.347(8) |
|  | Sn2 | 1x | 3.339(5) |  | P1 | 1x | 3.452(8) |
|  | Na1 | 1x | 3.3826(0) |  | P2 | 1x | 3.54(1) |
|  | Na1 | 1x | 3.3831(0) |  | P1 | 1x | 3.64(1) |
| Na5 | P2 | 2x | 2.82(1) |  | Na16 | 1x | 3.73(6) |
|  | Na14 | 2x | 2.897(6) | Na15 | Na11 | 1x | 2.65(2) |
|  | Na13 | 2x | 2.931(7) |  | Na10 | 1x | 2.83(1) |
|  | Na12 | 2x | 3.15(1) |  | Na12 | 1x | 2.85(2) |
|  | P1 | 2x | 3.20(1) |  | Na7 | 1x | 2.882(6) |
|  | Na4 | 1x | 3.32(1) |  | Na3 | 1x | 2.9141(0) |
|  | Sn3 | 1x | 3.38(1) |  | Na6 | 1x | 2.9144(0) |
|  | Na10 | 2x | 3.38(1) |  | Sn2 | 1x | 2.924(3) |
| Na6 | Na15 | 1x | 2.9141(0) |  | P1 | 1x | 3.07(1) |
|  | Na15 | 1x | 2.9146(0) |  | Na9 | 1x | 3.11(1) |
|  | P4 | 2x | 3.01(1) |  | P3 | 1x | 3.30(1) |
|  | P3 | 2x | 3.10(1) |  | P3 | 1x | 3.39(1) |
|  | Na2 | 1x | 3.356(0) |  | Na16 | 1x | 3.40(6) |
|  | Na3 | 1x | 3.3564(0) |  | P4 | 1x | 3.44(1) |
|  | Na10 | 2x | 3.39(1) |  | P2 | 1x | 3.60(1) |
|  | Na9 | 2x | 3.52(1) |  | P3 | 1x | 3.61(1) |
|  | Na16 | 2x | 3.95(6) | Na16 | Na11 | 1x | 1.55(6) |
| Na7 | P2 | 2x | 2.88(1) |  | P4 | 1x | 2.55(5) |
|  | Na15 | 2x | 2.882(6) |  | Na9 | 1x | 2.82(6) |
|  | Na13 | 2x | 2.946(6) |  | P2 | 1x | 2.83(6) |
|  | P3 | 2x | 3.00(1) |  | Na9 | 1x | 2.83(6) |
|  | Na12 | 2x | 3.11(2) |  | P3 | 1x | 3.11(6) |
|  | Na9 | 2x | 3.17(2) |  | Na10 | 1x | 3.23(5) |
|  | Na3 | 1x | 3.32(1) |  | Na15 | 1x | 3.40(6) |
|  | Sn3 | 1x | 3.43(1) |  | Na8 | 1x | 3.50(6) |

**Table S8:** Bond angles of up to 4Å of SnP_4_ and NaP_4_ tetrahedra and NaP_6_ octahedra in HT-Na_8_SnP_4_ at RT.

| **Atom 1 – 2 - 3** | | | Angle [°] | **Atom 1 – 2 - 3** | | | Angle [°] |
| --- | --- | --- | --- | --- | --- | --- | --- |
| P4 | Sn1 | P4 | 106.9(4) | P2 | Na13 | P2 | 83.1(3) |
| P4 | Sn1 | P4 | 114.6(4) | P2 | Na13 | P3 | 93.0(3) |
| P1 | Sn2 | P1 | 118.3(4) | P2 | Na13 | P1 | 97.9(3) |
| P1 | Sn2 | P3 | 101.3(4) | P2 | Na13 | P4 | 175.6(3) |
| P1 | Sn2 | P3 | 109.9(4) | P2 | Na13 | P2 | 82.7(3) |
| P3 | Sn2 | P3 | 116.7(4) | P2 | Na13 | P3 | 175.3(3) |
| P2 | Sn3 | P2 | 110.0(4) | P2 | Na13 | P1 | 94.3(3) |
| P2 | Sn3 | P2 | 108.9(4) | P2 | Na13 | P4 | 98.2(3) |
| P2 | Sn3 | P2 | 109.4(4) | P2 | Na13 | P3 | 98.0(3) |
| P1 | Na1 | P1 | 107.7(3) | P2 | Na13 | P1 | 176.7(3) |
| P1 | Na1 | P1 | 112.9(3) | P2 | Na13 | P4 | 92.7(3) |
| P4 | Na2 | P4 | 93.9(3) | P3 | Na13 | P1 | 84.9(3) |
| P4 | Na2 | P4 | 113.3(3) | P3 | Na13 | P4 | 86.2(3) |
| P4 | Na2 | P4 | 122.2(3) | P1 | Na13 | P4 | 86.3(3) |
| P4 | Na2 | P4 | 122.2(3) | P3 | Na14 | P1 | 83.6(3) |
| P4 | Na2 | P4 | 113.3(3) | P3 | Na14 | P1 | 93.1(3) |
| P4 | Na2 | P4 | 93.9(3) | P3 | Na14 | P1 | 85.2(3) |
| P3 | Na3 | P3 | 100.8(4) | P3 | Na14 | P2 | 94.2(3) |
| P3 | Na3 | P3 | 112.1(4) | P3 | Na14 | P1 | 175.9(3) |
| P3 | Na3 | P3 | 115.7(4) | P1 | Na14 | P1 | 94.2(3) |
| P1 | Na4 | P1 | 98.9(3) | P1 | Na14 | P1 | 87.5(3) |
| P1 | Na4 | P1 | 109.5(3) | P1 | Na14 | P2 | 176.7(3) |
| P1 | Na4 | P1 | 120.5(3) | P1 | Na14 | P1 | 92.3(3) |
| P2 | Na5 | P2 | 96.0(4) | P1 | Na14 | P1 | 177.4(3) |
| P2 | Na5 | P1 | 114.8(4) | P1 | Na14 | P2 | 88.3(3) |
| P2 | Na5 | P1 | 110.0(4) | P1 | Na14 | P1 | 86.3(3) |
| P4 | Na6 | P4 | 109.2(3) | P1 | Na14 | P2 | 89.7(3) |
| P4 | Na6 | P3 | 113.2(3) | P1 | Na14 | P1 | 95.4(2) |
| P4 | Na6 | P3 | 104.7(3) | P2 | Na14 | P1 | 89.7(3) |
| P3 | Na6 | P3 | 111.7(3) | P1 | Na15 | P3 | 82.2(3) |
| P2 | Na7 | P2 | 94.2(4) | P1 | Na15 | P3 | 86.6(3) |
| P2 | Na7 | P3 | 108.2(4) | P1 | Na15 | P4 | 97.5(3) |
| P2 | Na7 | P3 | 115.2(4) | P1 | Na15 | P2 | 91.6(3) |
| P3 | Na7 | P3 | 114.0(4) | P1 | Na15 | P3 | 175.5(3) |
| P2 | Na8 | P2 | 96.0(5) | P3 | Na15 | P3 | 88.6(3) |
| P2 | Na8 | P4 | 109.8(6) | P3 | Na15 | P4 | 174.7(3) |
| P2 | Na8 | P4 | 117.3(6) | P3 | Na15 | P2 | 87.1(3) |
| P4 | Na8 | P4 | 106.8(5) | P3 | Na15 | P3 | 93.4(3) |
| P4 | Na9 | P3 | 117.7(6) | P3 | Na15 | P4 | 96.5(3) |
| P4 | Na9 | P4 | 89.4(5) | P3 | Na15 | P2 | 175.6(3) |
| P4 | Na9 | P2 | 115.0(7) | P3 | Na15 | P3 | 94.3(3) |
| P3 | Na9 | P4 | 110.9(7) | P4 | Na15 | P2 | 87.5(3) |
| P3 | Na9 | P2 | 114.3(5) | P4 | Na15 | P3 | 86.6(3) |
| P4 | Na9 | P2 | 105.5(5) | P2 | Na15 | P3 | 87.0(3) |
| P1 | Na10 | P4 | 117.0(6) | P4 | Na16 | P2 | 129.8(7) |
| P1 | Na10 | P2 | 112.1(5) | P4 | Na16 | P3 | 115.6(2) |
| P1 | Na10 | P3 | 94.5(4) | P2 | Na16 | P3 | 110.2(2) |
| P4 | Na10 | P2 | 116.6(5) |  |  |  |  |
| P4 | Na10 | P3 | 112.2(5) |  |  |  |  |
| P2 | Na10 | P3 | 100.6(5) |  |  |  |  |
| P1 | Na11 | P2 | 115.0(9) |  |  |  |  |
| P1 | Na11 | P4 | 115.7(9) |  |  |  |  |
| P1 | Na11 | P3 | 107.6(8) |  |  |  |  |
| P2 | Na11 | P4 | 107.5(8) |  |  |  |  |
| P2 | Na11 | P3 | 107.3(8) |  |  |  |  |
| P4 | Na11 | P3 | 102.5(8) |  |  |  |  |
| P2 | Na12 | P3 | 119.3(7) |  |  |  |  |
| P2 | Na12 | P1 | 120.4(7) |  |  |  |  |
| P2 | Na12 | P1 | 116.3(8) |  |  |  |  |
| P3 | Na12 | P1 | 94.8(8) |  |  |  |  |
| P3 | Na12 | P1 | 99.4(6) |  |  |  |  |
| P1 | Na12 | P1 | 102.3(6) |  |  |  |  |
| P2 | Na13 | P2 | 82.5(3) |  |  |  |  |

Coordination Polyhedra


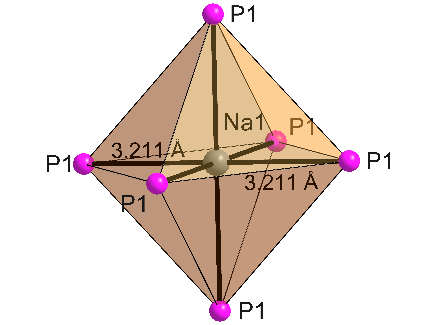

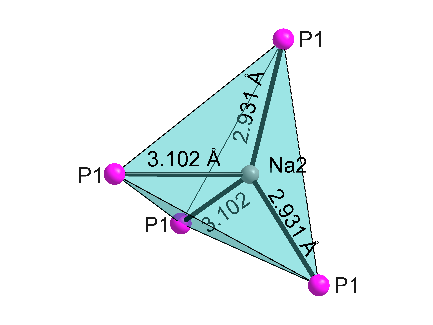

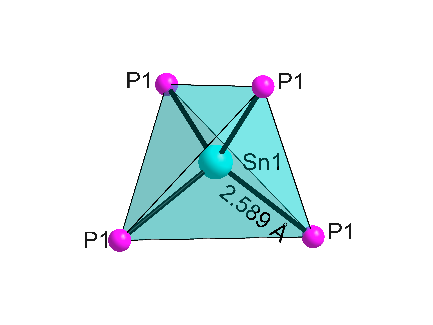


**Figure S3:** Coordination Polyhedra in LT-Na_8_SnP_4_. Octahedral coordination polyhedron of Na1 is depicted with orangefaces , tetrahedral coordination polyhedron of Na2 and Sn1 with turquoise color.


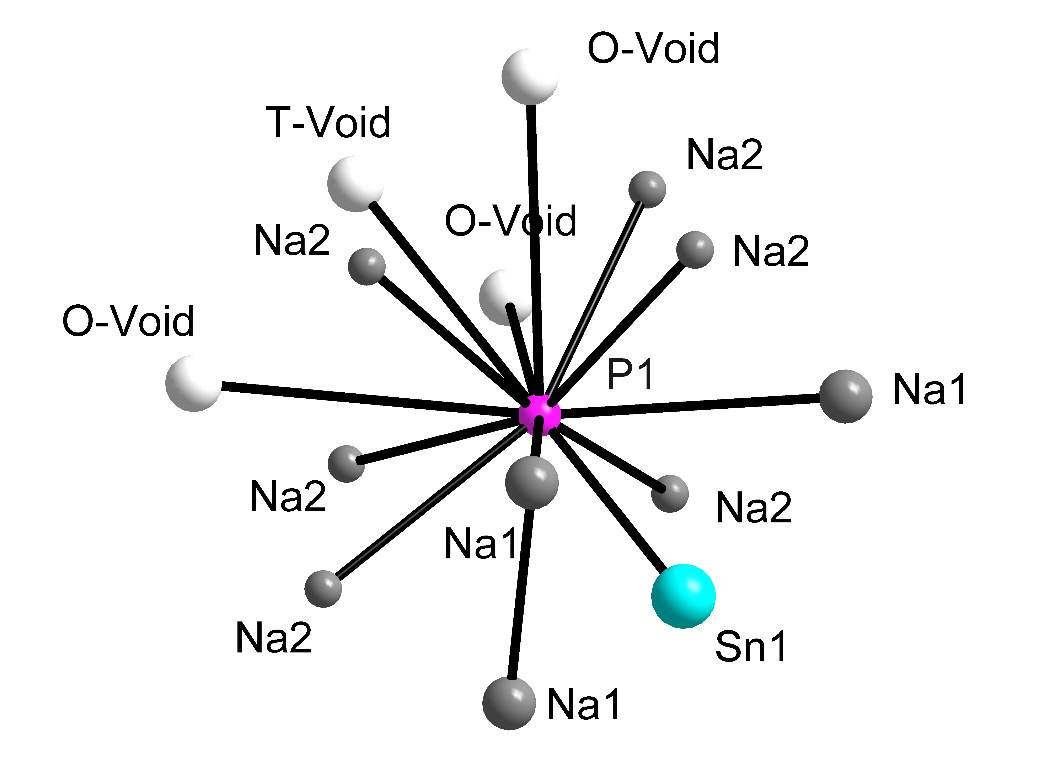


**Figure S4:** Coordination Sphere of phosphorous in LT-Na_8_SnP_4_. Occupied octahedral voids (Na1) are edge-sharing connected to the SnP_4_ tetrahedron. Unoccupied octahedral voids are connected to the unoccupied tetrahedral void.


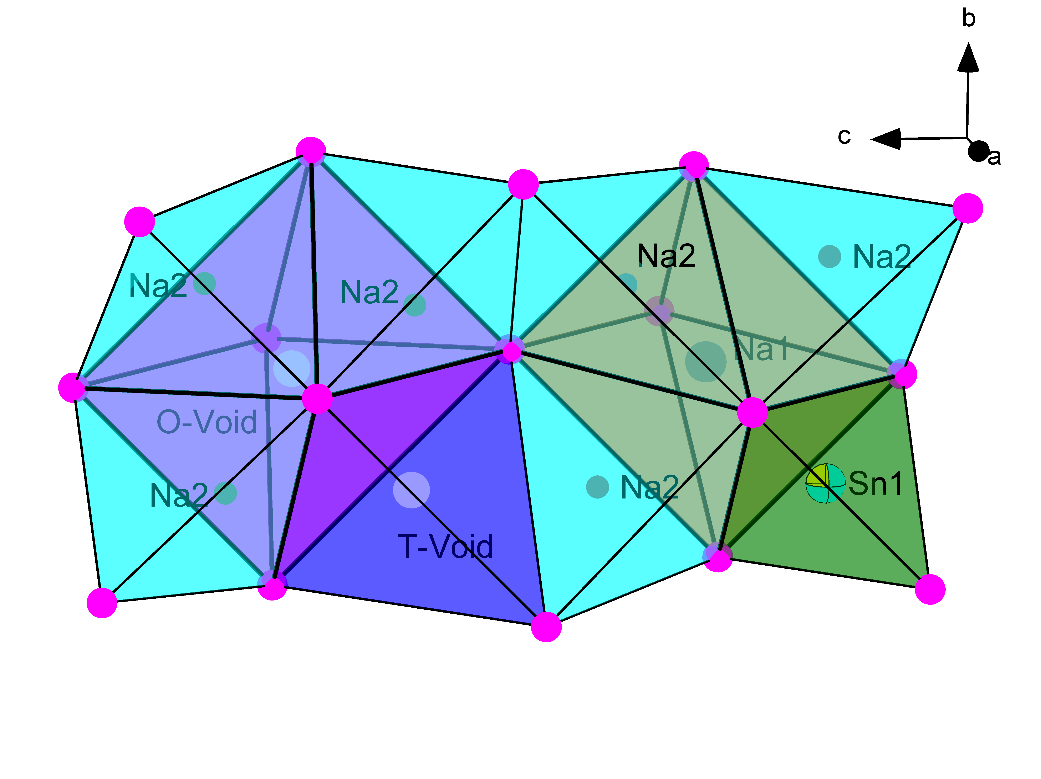

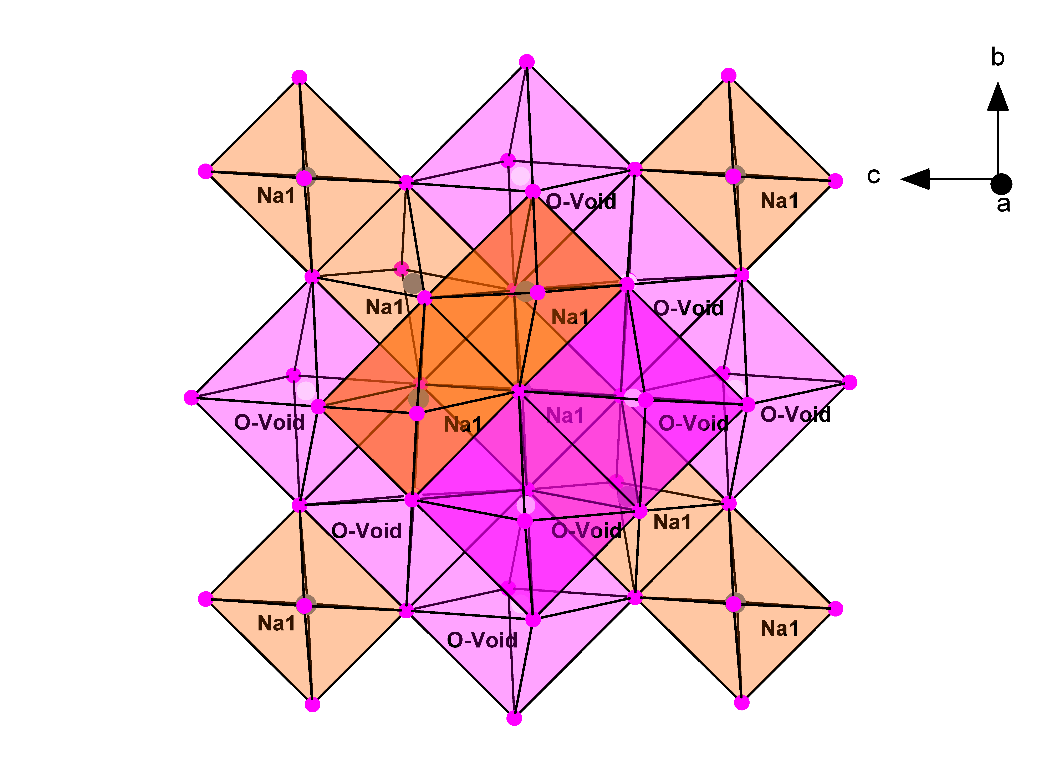


**Figure S5:** Interconnection between tetrahedral and octahedral voids in LT-Na_8_SnP_4_. a) Coordination network of all voids. Na1-octahedra are represented in orange, Na2-tetrahedra in turquoise, Sn-tetrahedra in green, empty tetrahedral voids in blue and empty octahedral voids in purple. b) Coordination network of only octahedral voids forming infinite edge-sharing strands of alternatingly occupied and unoccupied octahedra.


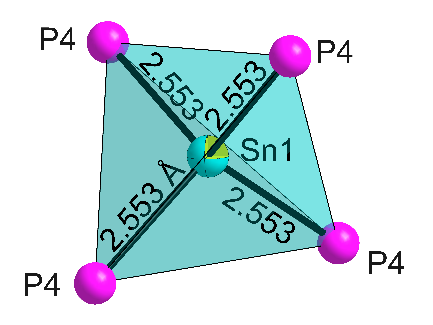

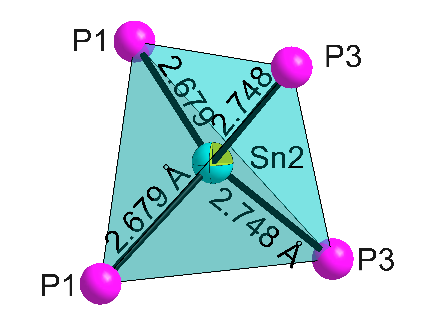

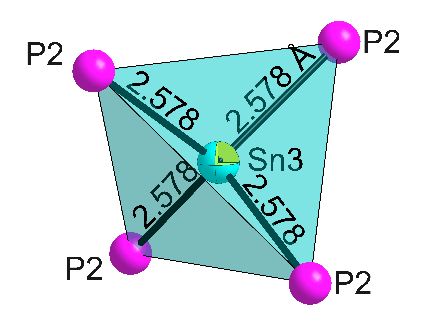

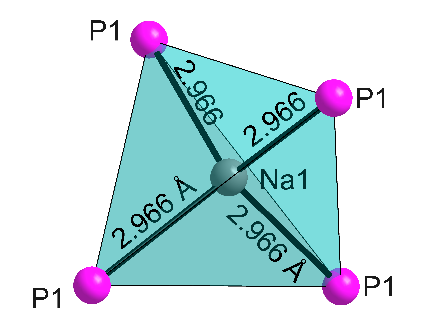

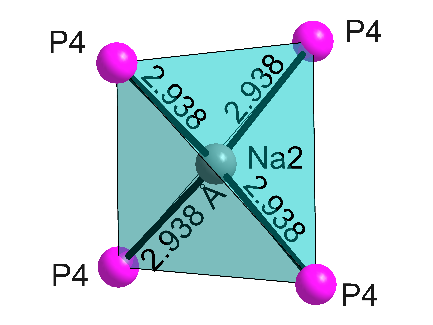

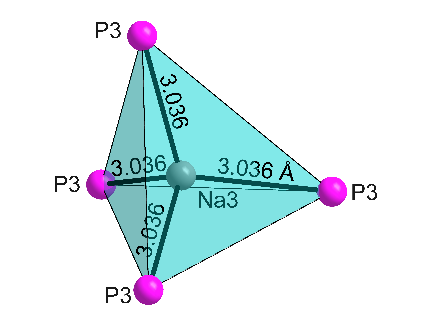

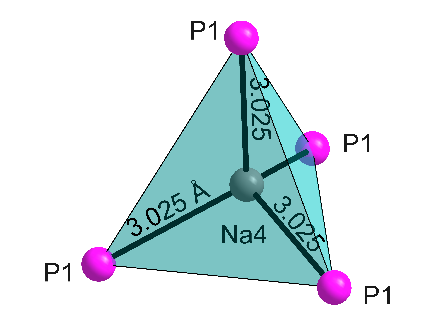

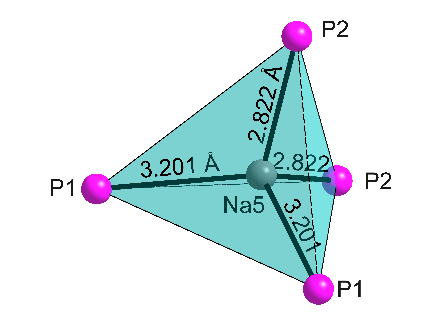

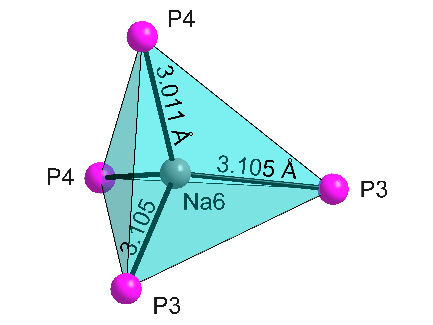

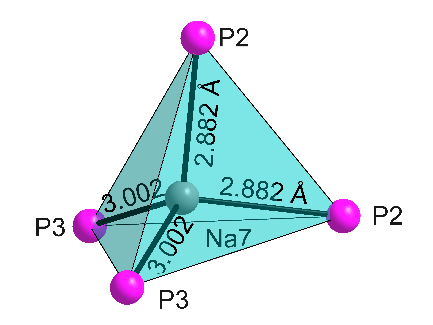

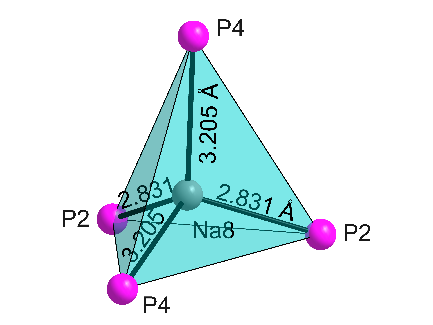

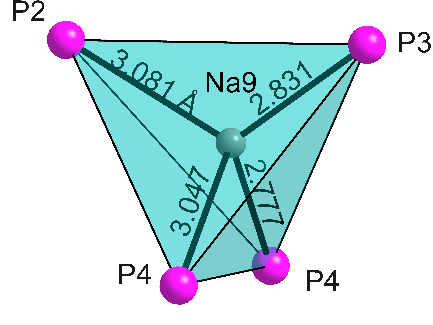

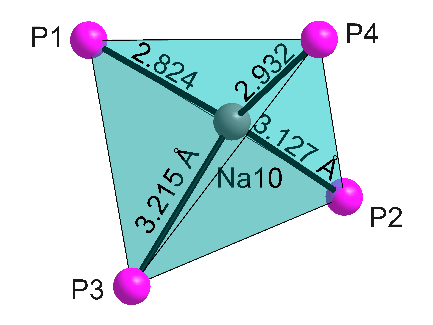

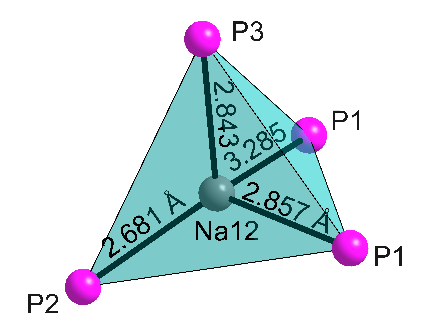

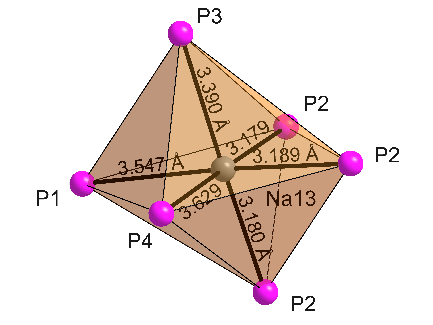

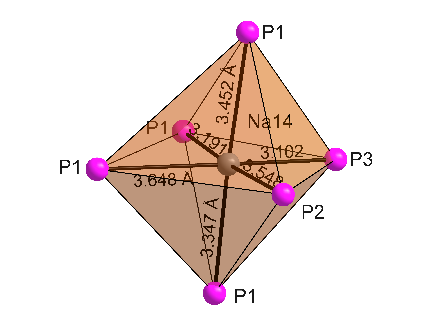

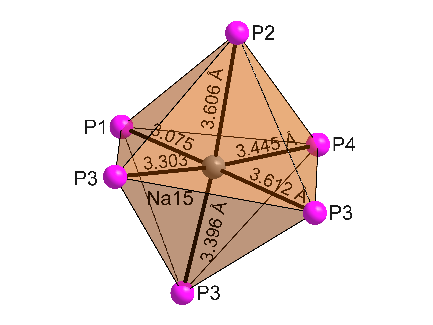


**Figure S6:** Coordination Polyhedra of Na and Sn in HT-Na_8_SnP_4_. Octahedral Sodium is depicted as orange polyhedra, tetrahedral sodium as turquoise and tin as green polyhedra, respectively.


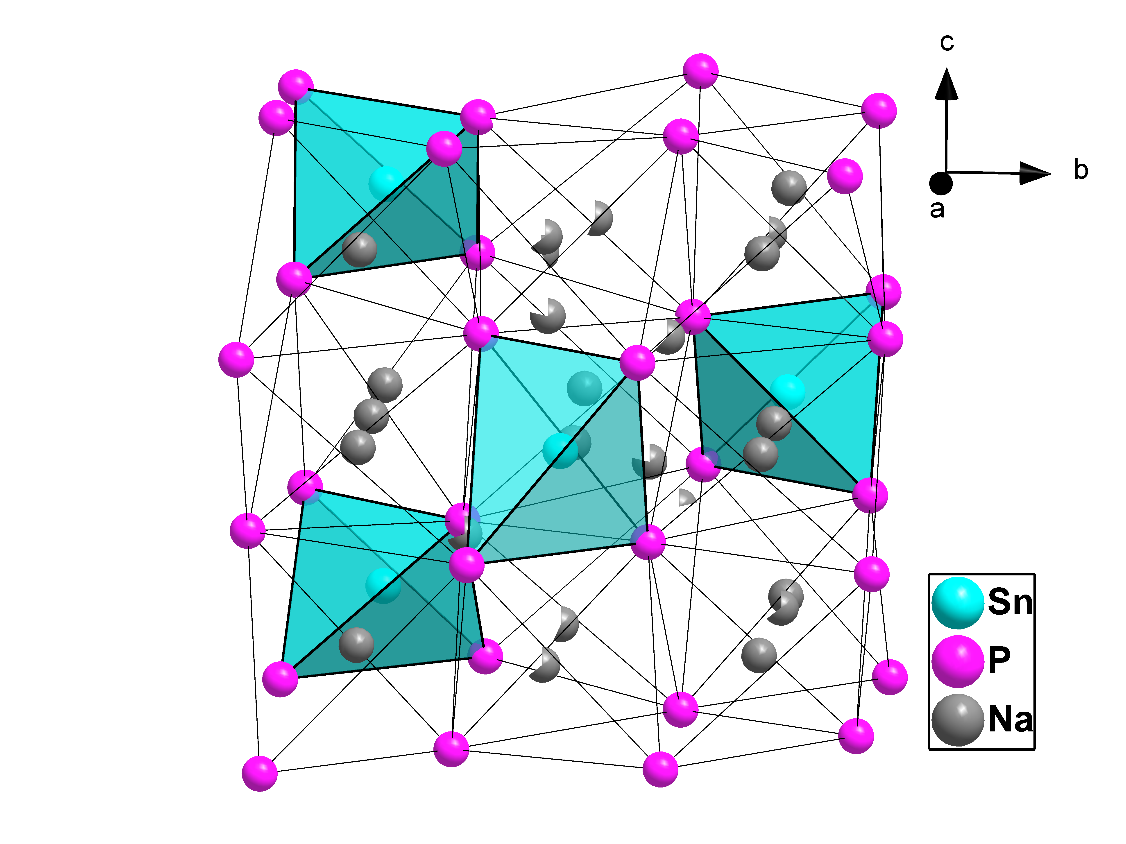


**Figure S7:** Asymmetric unit shown as simplified coordination network in HT-Na_8_SnP_4_. As all tetrahedral and octahedral voids are at least partially filled (Na-Positions are given in as partial spheres representing the occupation), the only breakpoint in an ion migration network are the Sn-filled tetrahedra, which show a comparably large repeating unit due to the tetragonal symmetry.

Bärnighausen Symmetry Degradation


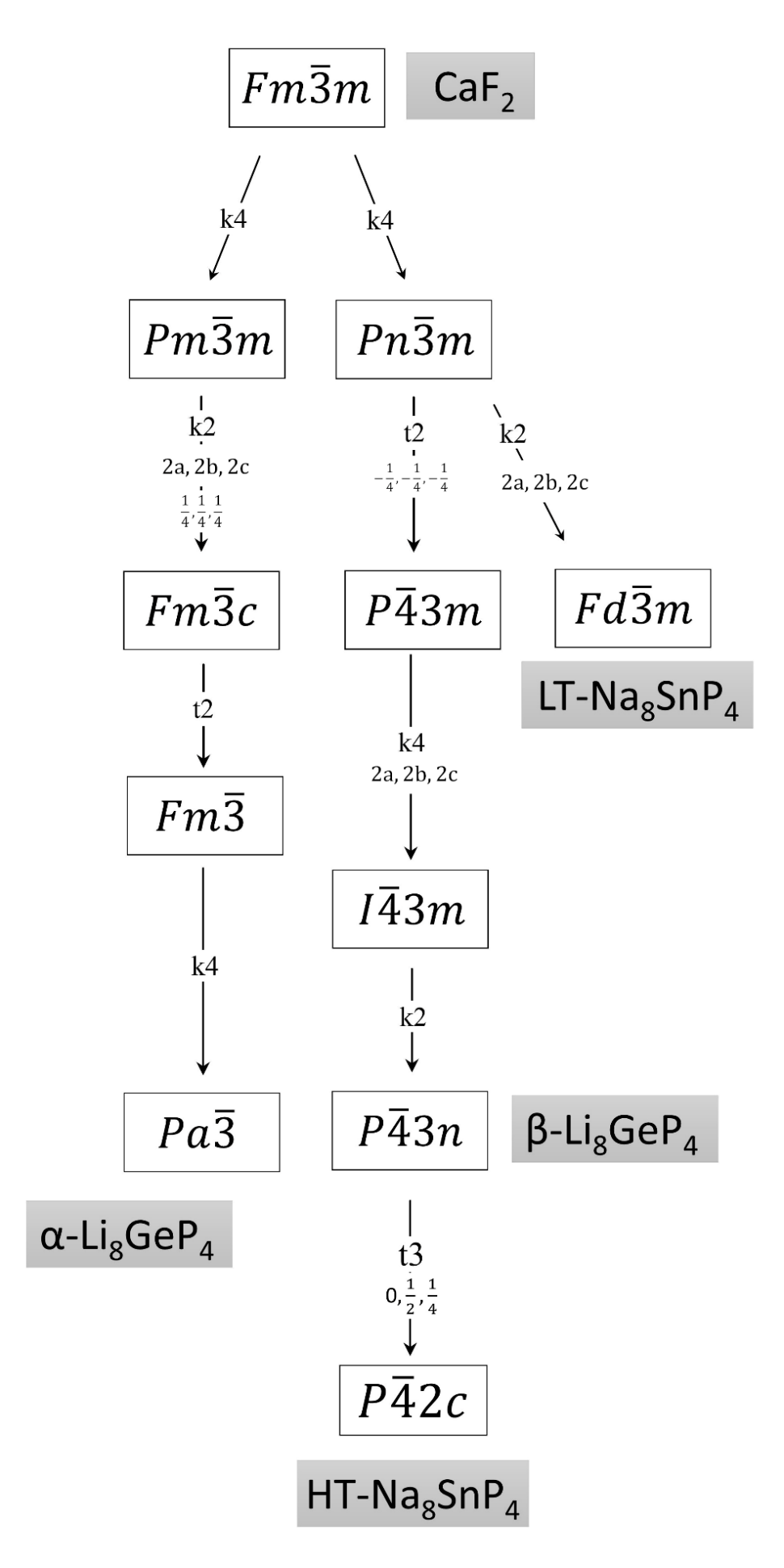


**Figure S8:** Symmetry relationship between CaF_2_ aristotype and the different phases of the alkali phosphidotetrelates shown in a Bärnighausen tree.

**Table S9:** Symmetry degradation according to Bärnighausen for LT-Na_8_SnP_4_ starting from CaF_2_.

| Fm$\bar{3}$m | Ca: 4*a* | F: 8*c* | □: 4*b* |
| --- | --- | --- | --- |
|  | *m*$\overline{\text{3}}$*m* | $\overline{\text{4}}$3*m* | *m*$\overline{\text{3}}$*m* |
|  | 1  1  1 | ^5^/_4_  ^5^/_4_  ^5^/_4_ | ^1^/_2_  ^1^/_2_  ^1^/_2_ |

| Pn$\bar{3}$m | 4*b* | 2*a* | 6*d* | 4*c* |
| --- | --- | --- | --- | --- |
|  | .$\bar{3}$*m* | $\overline{\text{4}}$3*m* | $\overline{\text{4}}$2.*m* | .$\bar{3}$*m* |
|  | 1  1  1 | ^5^/_4_  ^5^/_4_  ^5^/_4_ | ^3^/_4_  ^5^/_4_  ^1^/_4_ | ^1^/_2_  ^1^/_2_  ^1^/_2_ |

| Fd$\bar{3}$m | Na1:16*c* | □:16*d* | Sn1: 8*a* | □:8*b* | Na2: 48*f* | P1: 32*e* |
| --- | --- | --- | --- | --- | --- | --- |
|  | .$\bar{3}$*m* | .$\bar{3}$*m* | $\overline{\text{4}}$3*m* | $\overline{\text{4}}$3*m* | 2.*m* *m* | .$\bar{3}$*m* |
|  | 0  0  0 | ^1^/_2_  ^1^/_2_ ^1^/_2_ | ^1^/_8_  ^1^/_8_  ^1^/_8_ | ^3^/_8_  ^3^/_8_  ^3^/_8_ | 0.4003  ^1^/_8_  ^1^/_8_ | 0.23421  0.23421  0.23421 |

MEM Data


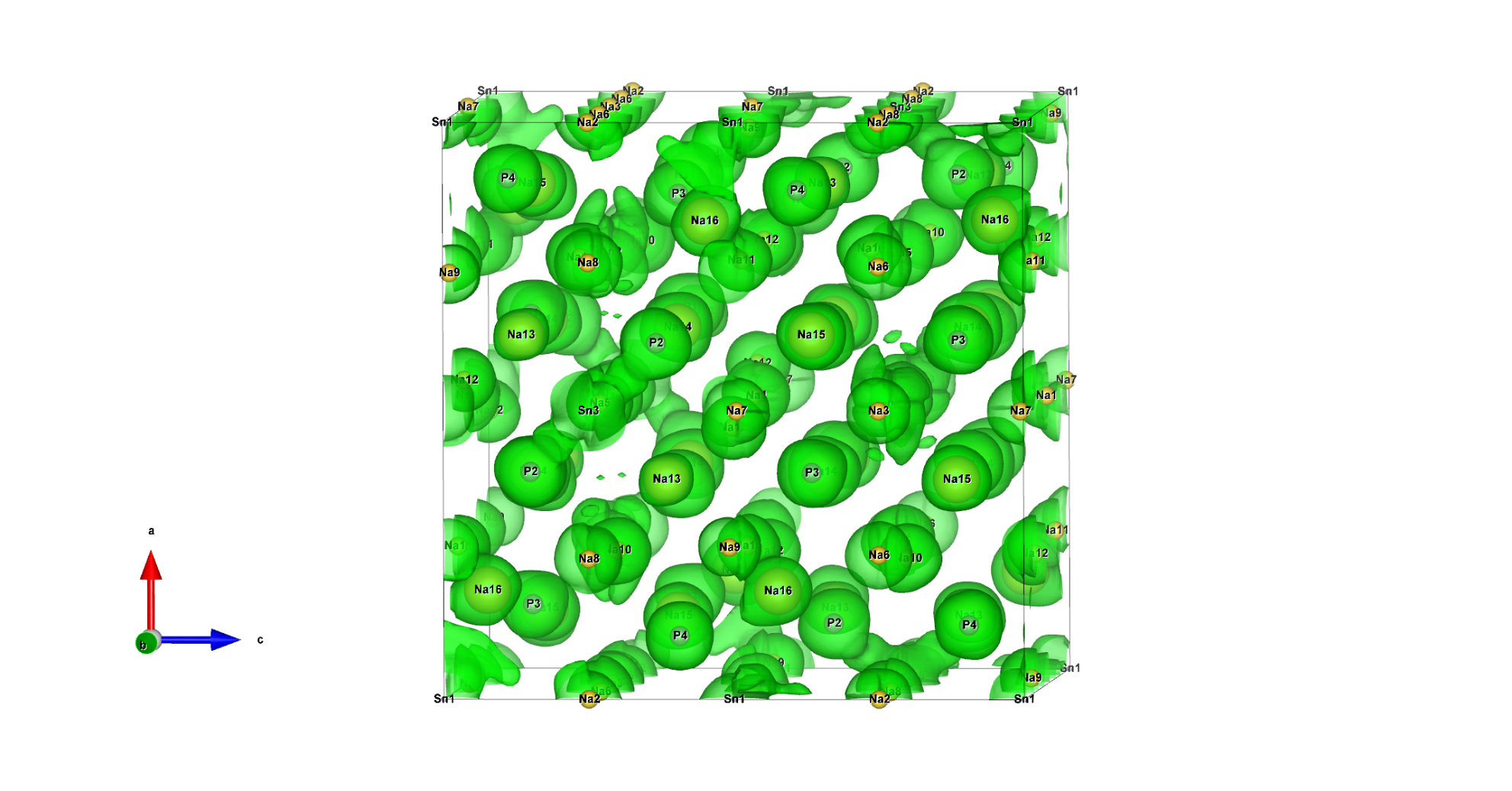


**Figure S9:** Full cell MEM electron charge density distributions of HT-Na_8_SnP_4_. Isosurfaces are given at a threshold of 0.5 eÅ^-3^. Atom ellipsoids are drawn at 50% probability level.

NMR Data


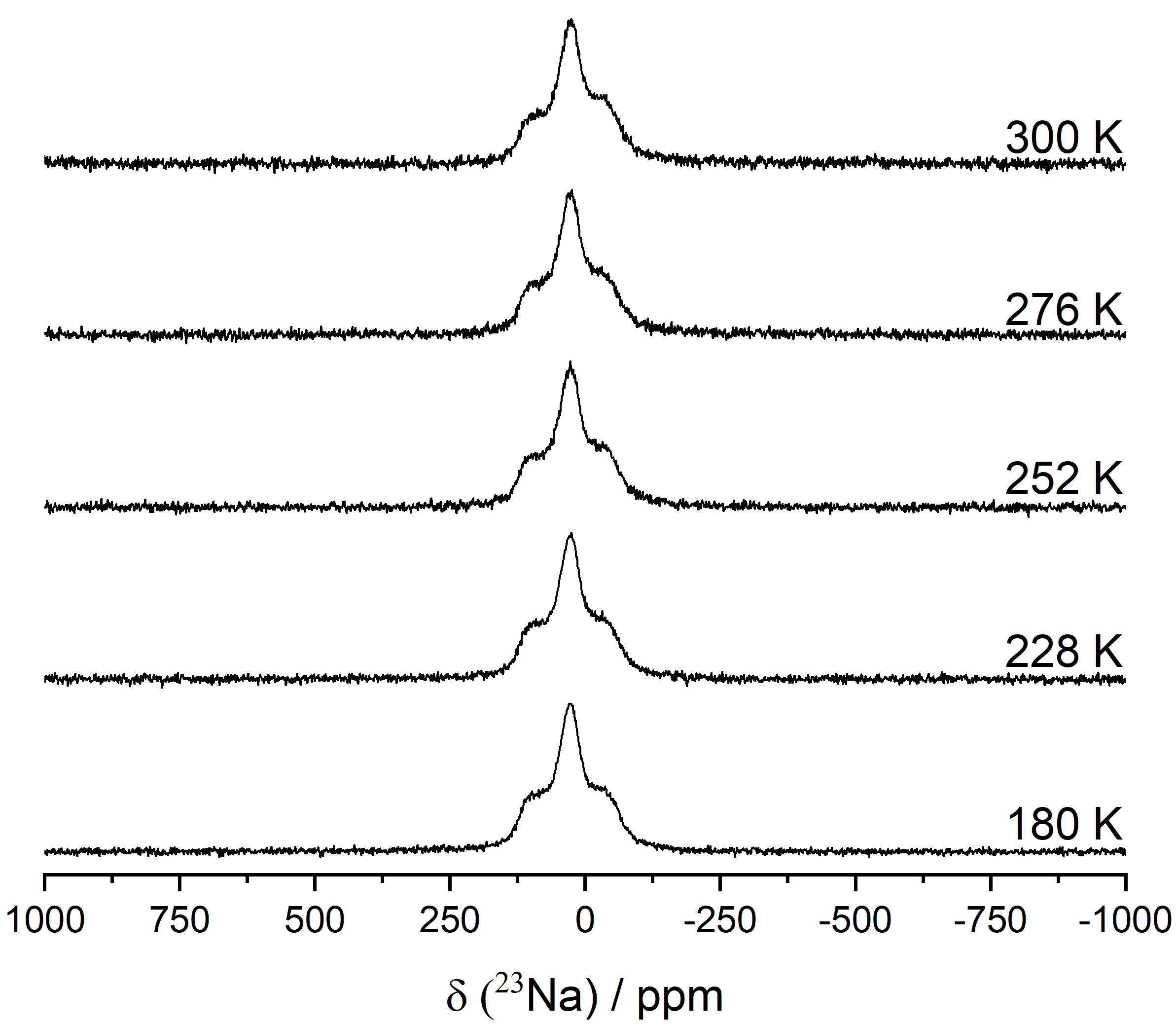

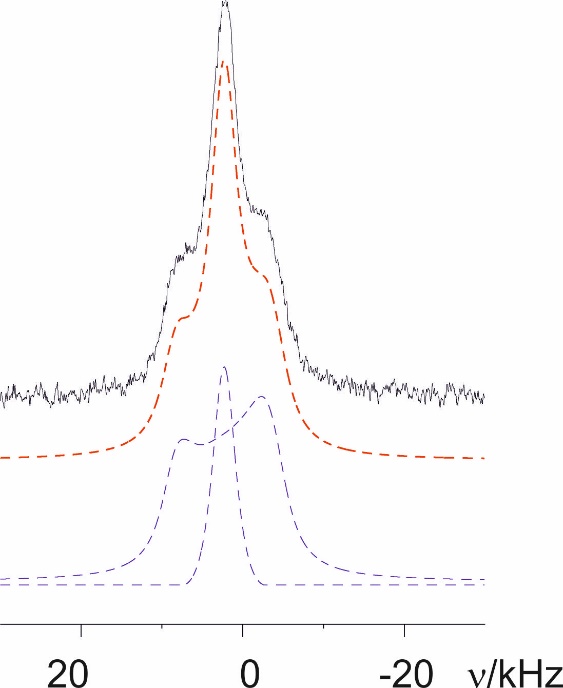


**Figure S10:** Static ^23^Na NMR measurement of LT-Na_8_SnP_4_ (left) and the fit of the corresponding signal (right)

**Table S10:** Fit data of ^23^Na static and MAS-NMR of LT-Na_8_SnP_4_ and the corresponding simulation using the program Wien2K^[13]^ and the given crystal structure of LT-Na_8_SnP_4_.

| **Peak** | **Method** | **Position [ppm]** | **C_Q_ [MHz]** | ***η*** | ***rel. area*** |  |  |
| --- | --- | --- | --- | --- | --- | --- | --- |
| Peak 1 | Static | 32 | 0.9 | 0 | 25 |  |  |
|  | MAS | 32 | 0.9 | 0 | 25 |  |  |
|  | Simulation Wien2K (Na1) |  | 0.7 | 0 |  |  |  |
| Peak 2 | Static | 52 | 2.8 | 0.1 | 75 |  |  |
|  | MAS | 48 | 2.4 | 0 | 75 |  |  |
|  | Simulation Wien2K (Na2) |  | 2.4 | 0 | -1.0∙10^21^ |  |  |

DC-Polarization curves

b)

a)

**Figure S11:** DC-Polarization curves of a) LT-Na_8_SnP_4_ and b) HT-Na_8_SnP_4_.

References

[1] STOE & Cie GmbH, Darmstadt, Germany, **2011**.

[2] A.-C. Dippel, H.-P. Liermann, J. T. Delitz, P. Walter, H. Schulte-Schrepping, O. H. Seeck, H. Franz, *J. Synch. Rad.* **2015**, *22*, 675-687.

[3] V. Dyadkin, P. Pattison, V. Dmitriev, D. Chernyshov, *J. Synch. Rad.* **2016**, *23*, 825-829.

[4] Institute Laue-Langevin, Grenoble, France, **2020**.

[5] P. Thompson, D. E. Cox, J. B. Hastings, *J. Appl. Crystallogr.* **1987**, *20*, 79-83.

[6] M. Botta, S. Zeitz, T. F. Fässler, *Z. Anorg. Allg. Chem.*, *n/a*, e202300166.

[7] A. A. Belik, T. Ikeda, F. Izumi, K. Momma, *Powder Diffr.* **2013**, *28*, 184-193.

[8] K. Momma, F. Izumi, *J. Appl. Crystallogr.* **2008**, *41*, 653-658.

[9] Netzsch-Gerätebau GmbH, Selb, **2006**.

[10] 14.2.0 ed., Mestrelab Research S.L., **2020**.

[11] D. Massiot, F. Fayon, M. Capron, I. King, S. Le Calvé, B. Alonso, J.-O. Durand, B. Bujoli, Z. Gan, G. Hoatson, *Magn. Reson. Chem.* **2002**, *40*, 70-76.

[12] A. Bielecki, D. P. Burum, *J. Magn. Reson., Ser A* **1995**, *116*, 215-220.

[13] a) P. Blaha, K. Schwarz, F. Tran, R. Laskowski, G. K. H. Madsen, L. D. Marks, *T. J. Chem. Phys.* **2020**, *152*; b) P. Blaha, K. Schwarz, G. K. Madsen, D. Kvasnicka, J. Luitz, *An augmented plane wave+ local orbitals program for calculating crystal properties* **2001**, *60*.
